# Risk of Infections and Cancer in Patients With Rheumatologic Diseases Receiving Interleukin Inhibitors: A Systematic Review and Meta-analysis

**DOI:** 10.1001/jamanetworkopen.2019.13102

**Published:** 2019-10-18

**Authors:** Jawad Bilal, Adam Berlinberg, Irbaz Bin Riaz, Warda Faridi, Sandipan Bhattacharjee, Gilbert Ortega, Mohammad H. Murad, Zhen Wang, Larry J. Prokop, Abdullah A. Alhifany, C. Kent Kwoh

**Affiliations:** 1 Division of Rheumatology, Department of Medicine, University of Arizona, Tucson; 2 Division of Rheumatology, Department of Medicine, University of Colorado, Denver; 3 Division of Hematology/Oncology, Department of Medicine, Mayo Clinic Rochester, Rochester, Minnesota; 4 Division of Hematology/Oncology, Department of Medicine, University of Arizona, Tucson; 5 College of Pharmacy, Department of Pharmacy Practice and Science, University of Arizona, Tucson; 6 Department of Medicine, University of Arizona, Tucson; 7 Evidence-Based Practice Center, Mayo Clinic Rochester, Rochester, Minnesota; 8 Mayo Clinic Libraries, Mayo Clinic Rochester, Rochester, Minnesota; 9 College of Pharmacy, Department of Clinical Pharmacy, Umm Al-Qura University, Makkah, Saudi Arabia; 10 University of Arizona Arthritis Center, University of Arizona, Tucson

## Abstract

**Importance:**

The safety profile of interleukin (IL) inhibitors is not well established.

**Objective:**

To assess the risk of serious infections, opportunistic infections, and cancer in patients with rheumatologic diseases treated with IL inhibitors.

**Data Sources:**

Ovid MEDLINE and Epub Ahead of Print, In-Process & Other Non-Indexed Citations; Ovid MEDLINE Daily; Ovid Embase; Ovid Cochrane Central Register of Controlled Trials; Ovid Cochrane Database of Systematic Reviews; and Scopus were searched (inception to November 30, 2018).

**Study Selection:**

Randomized, placebo-controlled trials that evaluated IL inhibitor therapies in rheumatic diseases and reported safety data were included in the analyses.

**Data Extraction and Synthesis:**

This systematic review is reported according to the Preferred Reporting Items for Systematic Reviews and Meta-analyses (PRISMA) statement. Two investigators independently extracted study data and assessed risk of bias and certainty in the evidence. Fixed-effects meta-analysis was conducted to pool odds ratios (ORs) for serious infections, opportunistic infections, and cancers for IL inhibitors vs placebo.

**Main Outcomes and Measures:**

The outcomes of interest were the number of serious infections, opportunistic infections, and cancers in individuals receiving IL inhibitor therapies compared with placebo.

**Results:**

In this meta-analysis, 74 studies comprising 29 214 patients (24 236 patients for serious infections, 9998 for opportunistic infections, and 21 065 for cancer [number of patients overlaps for each outcome]) were included. Patients receiving IL inhibitors had a higher risk of serious infections (OR, 1.97; 95% CI, 1.58-2.44; *P* < .001, *I*^2^ = 0%; high certainty), opportunistic infections (OR, 2.35; 95% CI, 1.09-5.05; *P* = .03, *I*^2^ = 0%; moderate certainty), and cancer (OR, 1.52; 95% CI, 1.05-2.19; *P* = .03, *I*^2^ = 11%; moderate certainty).

**Conclusions and Relevance:**

The risk of serious infections, opportunistic infections, and cancer appears to be increased in patients with rheumatologic diseases who are treated with IL inhibitors compared with placebo.

## Key Points

**Question:**

What is the risk of serious infections, opportunistic infections, and cancer in patients with rheumatologic diseases treated with interleukin inhibitors?

**Findings:**

In this systematic review and meta-analysis of 74 randomized clinical trials comprising 29 214 patients, pooled results suggest that risk of serious infections, opportunistic infections, and cancer is increased in patients with rheumatologic diseases who are treated with interleukin inhibitors compared with placebo.

**Meaning:**

This analysis suggests estimates of risk for infections and cancer associated with the use of interleukin inhibitors that can inform shared decision-making when patients and clinicians are contemplating the use of interleukin inhibitors for rheumatologic diseases.

## Teaser

This systematic review and meta-analysis examines the risk for serious infections, opportunistic infections, and cancers in individuals receiving interleukin inhibitor therapies for rheumatologic diseases.

## Introduction

Interleukins (ILs) are cytokines that play a central role in immune regulation and inflammation by promoting proliferation, activation, migration, and regulation of leukocytes.^1^ Therefore, several ILs have been targeted for treatment of immunologic diseases, including rheumatoid arthritis, psoriasis, psoriatic arthritis, ankylosing spondylitis, and inflammatory bowel disease. Interleukin-1 inhibitors (eg, anakinra, rilonacept), IL-6 inhibitors (eg, tocilizumab, sarilumab), IL-12/23 inhibitors (eg, ustekinumab), and IL-17 inhibitors (eg, ixekizumab, secukinumab) have been approved for clinical use in rheumatologic diseases by the US Food and Drug Administration and by the European Medicines Agency.

Although the therapeutic efficacy of these targeted biologics is well established by several clinical trials, systematic reviews, and meta-analyses,^2,3,4,5,6,7^ there is a paucity of data regarding the safety profile of these agents. The increased risk of serious and opportunistic infections with biologics, including IL inhibitors, has been a plausible safety concern secondary to blockade of biological pathways leading to immune dysregulation.^7,8,9,10^ However, the currently available evidence is not sufficient to draw conclusions regarding the safety of IL inhibitors with regard to the risk of serious infections and cancer.^11^ Establishing the safety data for rare adverse events, such as serious infections and cancer, is challenging because individual clinical trials lack adequate sample size. Previous meta-analyses have successfully identified a significant incidence of rare adverse effects by pooling the data in similar situations in which critical toxic effect signals were missed when looking at individual trials.^12^ For example, a meta-analysis suggested that treatment with rosiglitazone was associated with a significant increase in the risk of myocardial infarction.^13^ Similarly, several systematic reviews and meta-analyses have attempted to define the safety of tumor necrosis factor (TNF) inhibitors and supported the risk profile by consistent research evidence.^14,15,16,17,18,19,20,21^

Despite widespread use, it is still uncertain to what extent therapy with IL inhibitors may be associated with an increased risk of serious infections and cancer. Therefore, we conducted a systematic review and meta-analysis of published clinical trial data to assess the risk of serious infections, opportunistic infections, and cancer in individuals treated with IL inhibitors for any indicated rheumatologic condition.

## Methods

This study followed the Preferred Reporting Items for Systematic Reviews and Meta-analyses (PRISMA) reporting guideline for systematic reviews and meta-analyses and was conducted following an a priori established protocol.

The Ovid MEDLINE and Epub Ahead of Print, In-Process & Other Non-Indexed Citations; Ovid MEDLINE Daily; Ovid Embase; Ovid Cochrane Central Register of Controlled Trials; Ovid Cochrane Database of Systematic Reviews; and Scopus were searched from inception to November 30, 2018. The search strategy was designed and conducted by an experienced librarian with input from the study’s principal investigator (J.B.). Controlled vocabulary supplemented with keywords was used to search for randomized placebo-controlled trials of IL inhibitor therapy for rheumatic diseases. A detailed search strategy is provided in the eAppendix in the Supplement.

Any randomized, placebo-controlled trials that evaluated IL inhibitor therapies in rheumatic diseases and reported serious infections, opportunistic infections, and/or cancers were included. Inhibitors of the following ILs were considered: anakinra (IL-1), rilonacept (IL-1), canakinumab (IL-1), tocilizumab (IL-6), olokizumab (IL-6), clazakizumab (IL-6), sirukumab (IL-6), sarilumab (IL-6), ustekinumab (IL-12/23), brodalumab (IL-17), secukinumab (IL-17), ixekizumab (IL-17), and guselkumab (IL-23). In studies with multiple intervention arms, data were extracted from the IL inhibitor arm. In case of multiple reports from the same study, the data obtained at the longest follow-up duration were considered.

Two of us (A.B., W.F.) screened the titles and abstracts independently; the full texts were screened if the articles met the inclusion criteria. Full text of these selected articles was obtained and evaluated by 2 of us (A.B., W.F.) to confirm eligibility for inclusion. Any discrepancy was resolved via discussion. If there was disagreement between the reviewers, a third investigator (J.B.) was contacted and a decision was made through discussion. Data were recorded in a standardized manner, including the last name of first author, year of publication, disease studied, number of patients in treatment and placebo arms, treatment drug, control drug, dosage of drug, follow-up duration, number of serious infections, number and type of opportunistic infections, and number and type of cancers.

The Cochrane Collaboration Risk of Bias Assessment Tool was used to assess for selection bias (random sequence generation, allocation concealment), performance bias (blinding of participant and personnel), detection bias (blinding of outcome assessment), attrition bias (incomplete data), reporting bias (selective reporting), and other sources of bias.^22^ Certainty of evidence was determined using the GRADE (grades of recommendation, assessment, development, and evaluation) approach.^23^

The outcomes of interest were the number of serious infections, opportunistic infections, and cancers in individuals receiving IL inhibitor therapies compared with placebo. The serious infections were predefined by study investigators using previously validated measures, as infections resulting in hospitalization, the use of antibiotics, or death. The definition of opportunistic infections was based on a consensus statement by Winthrop et al.^24^ Oropharyngeal candidiasis infections were grouped for analysis. Prespecified subgroup analyses were performed to evaluate the risk of serious infections with different IL inhibitor therapies and disease states.

### Statistical Analysis

The number of patients who received at least 1 dose of the IL inhibitor represented the denominator of our outcome measure. Fixed-effects meta-analysis was conducted to generate odds ratios (ORs) and 95% CIs. The fixed-effects analysis using the Mantel-Haenszel method was conducted because the studies’ estimates were weighted only according to their estimated variances and, therefore, it is more appropriate for pooling rare events.^25^ The continuity correction method suggested by Sweeting et al^26^ was used to adjust if no events were observed in 1 of the study arms, and studies were excluded from the primary analysis if there were no events in either of the study arms. The continuity correction for the treatment and control arm was 1/(R+1) and R/(R+1) respectively, where R is the ratio of control group to treatment group sizes. Sensitivity analysis was performed without continuity correction. Comprehensive Meta-Analysis, version 3 (Biosta) software was used for all data analysis.^27^

To estimate the absolute harm increase (number needed to harm [NNH]), we calculated and pooled risk differences from the included studies. The NNH equals the inverse of the pooled risk differences.

Meta-regression was performed using the fixed-effects model (method of moments) to explore heterogeneity and evaluate the association of the duration of treatment with the risk of adverse events. We converted all ORs by logarithmic transformation to achieve more symmetrical distributions. The natural logarithm of the OR was the dependent variable, and the duration of follow-up was entered as a covariate. We applied a weighted regression model so that the more precise studies have more influence in the analysis.

A cumulative meta-analysis was performed by adding individual studies chronologically, and the results were summarized as each new study was added. The purpose of this technique was to provide a visual presentation for the evolution of evidence over time and determine the point estimates. In addition, leave-1-out analysis was conducted by recalculating the pooled ORs while omitting 1 study in turn to assess the influence of single studies on the overall findings.

Heterogeneity was assessed using the *I*^2^ statistic that expressed the percentage of heterogeneity beyond what is expected by chance. The *I*^2^ values greater than 25% were consistent with a low degree of heterogeneity; 50%, moderate degree; and 75%, high degree of heterogeneity.^28^

Publication bias was assessed using funnel plots, and the Egger regression test with a 2-tailed *P* value less than .05 was considered to be statistically significant. If publication bias was detected, the Duval and Tweedie trim-and-fill method was used for adjustment.^29^

## Results

**Figure 1.**
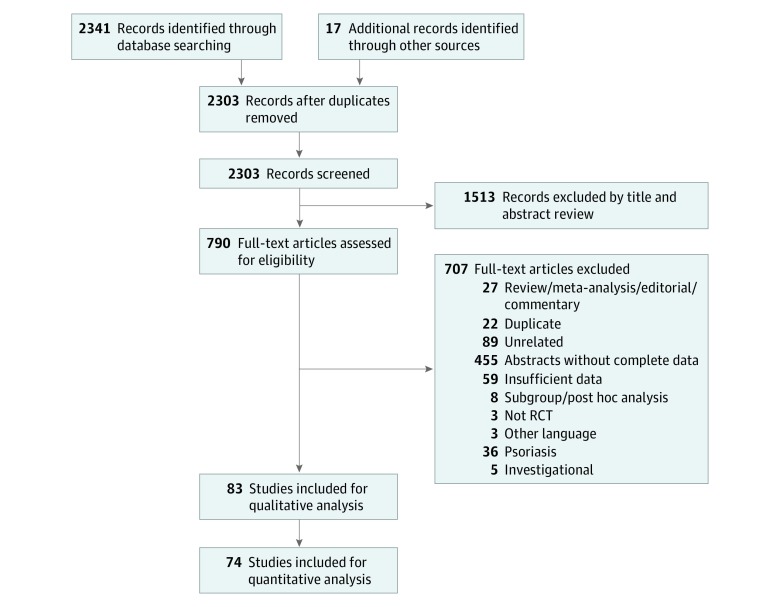
Preferred Reporting Items for Systematic Reviews and Meta-analyses (PRISMA) Flow Diagram RCT indicates randomized clinical trial.

A total of 2341 titles were retrieved using the initial database search; of these, 2303 studies were selected after removing duplicates, and 790 studies were considered eligible for further review after reviewing titles and abstracts. A total of 74 randomized clinical trials including 29 214 patients were found to have outcomes of interest^2,30,31,32,33,34,35,36,37,38,39,40,41,42,43,44,45,46,47,48,49,50,51,52,53,54,55,56,57,58,59,60,61,62,63,64,65,66,67,68,69,70,71,72,73,74,75,76,77,78,79,80,81,82,83,84,85,86,87,88,89,90,91,92,93,94,95,96,97,98,99,100,101,102,103,104,105,106,107,108,109^ (Figure 1). The characteristics of all of the included trials are described in the Table. Tocilizumab was evaluated in 18 trials, secukinumab in 15, anakinra in 8, ixekizumab in 6, rilonacept in 6, sarilumab in 4, sirukumab in 4, ustekinumab in 4, brodalumab in 3, guselkumab in 2, clazakizumab in 2, canakinumab in 1, and olokizumab in 1. There were 35 trials for rheumatoid arthritis, 12 for psoriatic arthritis, 9 for ankylosing spondylitis, 5 for gout, 5 for juvenile idiopathic arthritis, 2 for giant cell arteritis, 2 for systemic lupus erythematosus, 1 for primary Sjögren syndrome, 1 for systemic sclerosis, 1 for familial Mediterranean fever, and 1 for osteoarthritis.

**Table.** Studies Identified Using PRISMA Outcomes of Interest

Source	Disease Treated	Treatment Group (No. of Patients)	Placebo Group, No. of Patients	Follow-up, wk
Aletaha et al,^2^ 2017 (SIRROUND-T)	Rheumatoid arthritis	Sirukumab, 100 mg, every 2 wk (418); sirukumab, 50 mg, every 4 wk (416)	294	52
Alten et al,^30^ 2011	Rheumatoid arthritis	Canakinumab, 150 mg, every 4 wk (69); canakinumab, 300 mg, every 2 wk (64); canakinumab, 900 mg, every 2 wk (71)	70	12
Baek et al,^31^ 2019	Rheumatoid arthritis	Tocilizumab, 8 mg/kg, every 4 wk (89)	51	72
Baeten et al,^32^2013	Ankylosing spondylitis	Secukinumab, 10 mg/kg (24)	6	28
Baeten et al,^33^ 2015; Braun et al,^38^ 2017; Baraliakos et al,^35^ 2018 (MEASURE 1)	Ankylosing spondylitis	Secukinumab, 150 mg, every 4 wk (181); secukinumab, 75 mg, every 4 wk (179)	122	156
Baeten et al,^33^ 2015; Marzo-Ortega et al,^66^ 2017 (MEASURE 2)	Ankylosing spondylitis	Secukinumab, 150 mg (106); secukinumab, 75 mg (105)	74	104
Bao et al,^34^ 2011	Rheumatoid arthritis	Anakinra, 80 mg/d, with methotrexate (42)	12	24
Ben-Zvi et al,^36^ 2017	Familial Mediterranean fever	Anakinra, 100 mg/d (12)	13	16
Bijlsma et al,^37^ 2016	Rheumatoid arthritis	Tocilizumab, 8 mg/kg, every 4 wk with methotrexate (106); tocilizumab, 8 mg/kg, every 4 wk (103)	108	104
Brunner et al,^39^ 2015	Systemic juvenile idiopathic arthritis	Tocilizumab, 8 mg/kg (66); tocilizumab, 10 mg/kg (16)	81	24
Burmester et al,^40^ 2017 (FUNCTION)	Rheumatoid arthritis	Tocilizumab, 8 mg/kg, with methotrexate (527); tocilizumab, 8 mg/kg, with placebo (292); tocilizumab, 4 mg/kg, with methotrexate (289)	282	104
Chevalier et al,^41^ 2009	Osteoarthritis	Anakinra, 50 mg (34); anakinra, 150 mg (67)	69	12
Cohen et al,^42^ 2002	Rheumatoid arthritis	Anakinra, 0.04 mg/kg/d, with methotrexate (63); anakinra, 0.1 mg/kg/d, with methotrexate (74); anakinra, 0.4 mg/kg/d, with methotrexate (77); anakinra, 1.0 mg/kg/d, with methotrexate (59); anakinra, 2.0 mg/kg/d, with methotrexate (72)	74	24
Cohen et al,^43^ 2004	Rheumatoid arthritis	Anakinra, 100 mg/d, subcutaneously with methotrexate (250)	251	24
De Benedetti et al,^44^ 2012	Systemic juvenile idiopathic arthritis	Tocilizumab, 8-12 mg/kg (75)	37	12
Deodhar et al,^45^ 2018	Psoriatic arthritis	Guselkumab, 100 mg, every 8 wk (129)	49	56
Deodhar et al,^46^ 2019 (COAST-W)	Ankylosing spondylitis	Ixekizumab, 80 mg every 2 wk (98); ixekizumab, 80 mg, every 4 wk (114)	104	52
Emery et al,^108^ 2008	Rheumatoid arthritis	Tocilizumab, 4 mg/kg, with methotrexate (163); tocilizumab, 8 mg/kg, with methotrexate (175)	160	24
Fleischmann et al,^48^ 2003	Rheumatoid arthritis	Anakinra, 100 mg (1116)	283	24
Fleischmann et al,^47^ 2017 (TARGET)	Rheumatoid arthritis	Sarilumab, 150 mg, every 2 wk with csDMARDs (181); sarilumab, 200 mg, every 2 wk with csDMARDs (184)	181	24
Genovese et al,^53^ 2008	Rheumatoid arthritis	Tocilizumab, 8 mg/kg (802)	414	24
Genovese et al,^109^ 2010	Rheumatoid arthritis	Ixekizumab, 0.06-2.0 mg/kg (65)	22	24
Genovese et al,^50^ 2014 (1)	Rheumatoid arthritis	Tocilizumab, 8 mg/kg, every 4 wk (43); olokizumab, 60, 120, 240 mg, every 4 wk (23, 23, 22); olokizumab, 60, 120, 240 mg, every 2 wk (20, 22, 23)	55	12
Genovese et al,^49^ 2014 (2)	Rheumatoid arthritis	Secukinumab, 25, 75, 150, 300 mg, every 4 wk (54, 49, 43, 41)	50	60
Genovese et al,^52^ 2014 (3)	Rheumatoid arthritis	Biologic naive: ixekizumab, 3, 10, 30, 80, 180 mg (40, 35, 37, 57, 37);previous TNF use: ixekizumab, 80, 180 mg (65, 59)	54	12
Genovese et al,^51^ 2015 (MOBILITY)	Rheumatoid arthritis	Sarilumab, 150 mg, every 2 wk with methotrexate (431); sarilumab, 200 mg, every 2 wk with methotrexate (424)	427	52
Hueber et al,^106^ 2010	Rheumatoid arthritis	Secukinumab, 10 mg/kg, once (26)	26	12
Huizinga et al,^54^ 2014	Rheumatoid arthritis	Sarilumab, 100 mg, every 2 wk with methotrexate (51); sarilumab, 150 mg, every 2 wk with methotrexate (51); sarilumab, 100 mg, weekly with methotrexate (50); sarilumab, 200 mg, every 2 wk with methotrexate (52); sarilumab, 150 mg, weekly with methotrexate (50)	52	12
Ilowite et al,^55^ 2009	Juvenile rheumatoid arthritis	Anakinra, 1.0 mg/kg/d (25)	25	64
Khanna et al,^57^ 2016; Khanna et al,^58^ 2018 (faSScinate)	Systemic sclerosis	Tocilizumab, 162 mg, weekly (74)	44	96
Kivitz et al,^59^ 2014	Rheumatoid arthritis	Tocilizumab, 162 mg, every 2 wk (437)	218	24
Kivitz et al,^60^ 2018 (MEASURE 4)	Ankylosing spondylitis	Secukinumab, 150 mg, every 4 wk with loading dose (116); secukinumab, 150 mg, every 4 wk (117)	117	104
Kremer et al,^61^ 2011; Kremer et al,^62^ 2016	Rheumatoid arthritis	Tocilizumab, 4-8 mg/kg, with methotrexate (1149)	392	264
Lovell et al,^63^ 2013	Systemic juvenile idiopathic arthritis	Rilonacept, 2.2 mg/kg, or 4.4 mg/kg (23)	7	96
Maini et al,^64^ 2006	Rheumatoid arthritis	Tocilizumab, 2, 4, 8 mg/kg (53, 54, 52); tocilizumab, 2,4, 8 mg/kg, with methotrexate (52, 49, 50)	49	20
Martin et al,^65^ 2013	Rheumatoid arthritis	Brodalumab, 50, 140, 210 mg, every 2 wk (6, 6, 6); brodalumab, 420, 700 mg, every 4 wk (6, 6)	10	12
McInnes et al,^67^ 2013	Psoriatic arthritis	Ustekinumab, 45 mg (205); ustekinumab, 90 mg (204), given wk 0 and 4, and then every 12 wk	206	52
McInnes et al,^70^ 2014	Psoriatic arthritis	Secukinumab, 10 mg/kg, every 3 wk (28)	14	24
McInnes et al,^69^ 2017; McInnes et al,^68^2015 (FUTURE 2)	Psoriatic arthritis	Secukinumab, 75 mg, every 4 wk (99); secukinumab, 150 mg, every 4 wk (143); secukinumab, 300 mg, every 4 wk (145)	98	104
Mease et al,^72^ 2014	Psoriatic arthritis	Brodalumab, 140 mg (56); brodalumab, 280 mg (56)	55	52
Mease et al,^75^ 2015; Mease et al,^74^ 2018; Kavanaugh et al,^56^ 2017 (FUTURE 1)	Psoriatic arthritis	Secukinumab, 75 mg, every 4 wk (292); secukinumab, 150 mg, every 4 wk (434)	202	144
Mease et al,^73^ 2016 (1)	Psoriatic arthritis	Clazakizumab, 25 mg, every 4 wk with methotrexate (41); clazakizumab, 100 mg every 4 wk with methotrexate (42); clazakizumab, 200 mg, every 4 wk with methotrexate (41)	41	24
Mease et al,^76^ 2017 (2)	Psoriatic arthritis	Ixekizumab, 80 mg, every 2 wk (102); ixekizumab, 80 mg, every 4 wk (107); adalimumab, 40 mg (101)	106	24
Mease et al,^71^ 2018 (FUTURE 5)	Psoriatic arthritis	Secukinumab, 150 mg, every 4 wk with loading dose (220); secukinumab, 150 mg, every 4 wk (222); secukinumab, 300 mg, every 4 wk with loading dose (222)	332	24
Mitha et al,^77^ 2013	Gout	Rilonacept, 80 mg, weekly (82); rilonacept, 160 mg, weekly (84)	82	16
Nash et al,^78^ 2017	Psoriatic arthritis	Ixekizumab, 80 mg, every 2 wk (122); ixekizumab, 80 mg, every 4 wk (123)	118	24
Nash et al,^79^ 2018	Psoriatic arthritis	Secukinumab, 300 mg, every 4 wk (204); secukinumab, 150 mg, every 4 wk (202)	137	52
Nishimoto et al,^80^ 2009 (SATORI)	Rheumatoid arthritis	Tocilizumab, 8 mg/kg, every 4 wk (61)	64	24
Norheim et al,^81^ 2012	Primary Sjögren syndrome	Anakinra, 100 mg/d (13)	13	4
Pavelka et al,^82^ 2015	Rheumatoid arthritis	Brodalumab, 70 mg (63); brodalumab, 140 mg (63); brodalumab, 210 mg (63)	63	12
Pavelka et al,^83^ 2017 (MEASURE 3)	Ankylosing spondylitis	Secukinumab, 300 mg, every 4 wk (113); secukinumab, 150 mg, every 4 wk (110)	75	52
Quartier et al,^84^ 2011	Juvenile rheumatoid arthritis	Anakinra, 2 mg/kg/d (12)	12	52
Ritchlin et al,^85^ 2014	Psoriatic arthritis	Ustekinumab, 45 mg (134); ustekinumab, 90 mg (104), given wk 0, 4, and then every 12 wk	104	60
Rovin et al,^86^ 2016	Lupus nephritis	Sirukumab, 10 mg/kg (21)	4	24
Schumacher et al,^88^ 2012 (phase 2)	Gout	Rilonacept, 160 mg, weekly (41)	42	60
Schumacher et al,^87^2012 (phase 3)	Gout	Rilonacept, 80 mg, weekly (80); rilonacept, 160 mg, weekly (81)	79	20
Scott et al,^107^ 2016	Rheumatoid arthritis	Anakinra, 100 mg/d, with methotrexate (79)	75	104
Sieper et al,^91^ 2014	Ankylosing spondylitis	Tocilizumab, 8 mg/kg, every 4 wk (51)	51	12
Sieper et al,^89^ 2015 (ALIGN)	Ankylosing spondylitis	Sarilumab, 100 mg, every 2 wk (49); sarilumab, 150 mg, every 2 wk with methotrexate (50); sarilumab, 100 mg, weekly (52); sarilumab, 200 mg, every 2 wk (50); sarilumab, 150 mg, weekly (50)	50	12
Sieper et al,^90^2017	Ankylosing spondylitis	Secukinumab, 75 mg, every 4 wk (73); secukinumab, 150 mg, every 4 wk (72)	74	52
Smolen et al,^93^ 2008	Rheumatoid arthritis	Tocilizumab, 4 mg/kg, every 4 wk with methotrexate (212); tocilizumab, 8 mg/kg, every 4 wk with methotrexate (206)	204	32
Smolen et al,^94^ 2014	Rheumatoid arthritis	Sirukumab, 100 mg, every 2 wk (56); sirukumab, 100 mg, every 4 wk (30); sirukumab, 50 mg, every 4 wk (30); sirukumab, 25 mg, every 4 wk (31)	30	38
Smolen et al,^92^2017	Rheumatoid arthritis	Guselkumab, 50 mg, every 8 wk with methotrexate (55); guselkumab, 200 mg, every 8 wk with methotrexate (54); ustekinumab, 90 mg, every 12 wk with methotrexate (55); ustekinumab, 90 mg, every 8 wk with methotrexate (54)	55	48
Stone et al,^95^ 2017	Giant cell arteritis	Tocilizumab, 162 mg, weekly with 26-wk prednisone taper (100); tocilizumab, 162 mg, every 2 wk with 26-wk prednisone taper (49)	50	52
Sundy et al,^96^ 2014	Gout	Rilonacept, 160 mg, weekly (985)	330	20
Tahir et al,^97^ 2017	Rheumatoid arthritis	Secukinumab, 150 mg, every 4 wk (125); secukinumab, 75 mg, every 4 wk (124)	214	104
Takeuchi et al,^98^ 2017	Rheumatoid arthritis	Sirukumab, 100 mg, every 2 wk (662); sirukumab, 50 mg, every 4 wk (663)	556	52
Terkeltaub et al,^99^ 2013	Gout	Rilonacept, 320 mg, with oral placebo (75); rilonacept, 320 mg, with oral indomethacin (74)	76	4
Tlustochowicz et al,^100^ 2016	Rheumatoid arthritis	Secukinumab intravenous loading dose followed by 150 mg subcutaneously, every 4 wk (88); secukinumab intravenous loading dose followed by 150 mg subcutaneously, every 4 wk (89)	44	16
van der Heijde et al,^101^ 2018	Rheumatoid arthritis	Ixekizumab, 80 mg, every 2 wk (83); ixekizumab, 80 mg, every 4 wk (81)	87	24
van Vollenhoven et al,^102^2018	Systemic lupus erythematosus	Ustekinumab, 90 mg, every 8 wk (60)	42	24
Villiger et al,^103^ 2016	Giant cell arteritis	Tocilizumab, 8 mg/kg, every 4 wk with prednisolone (20)	10	52
Weinblatt et al,^104^ 2015	Rheumatoid arthritis	Clazakizumab, 25 mg, every 4 wk with methotrexate (59); clazakizumab, 100 mg, every 4 wk with methotrexate (60); clazakizumab, 200 mg, every 4 wk with methotrexate (60); clazakizumab, 100 mg, every 4 weeks with placebo (60); clazakizumab, 200 mg, every 4 wk with placebo (59); adalimumab, 40 mg, with methotrexate (59)	61	24
Yazici et al,^105^ 2012	Rheumatoid arthritis	Tocilizumab, 8 mg/kg, every 4 wk with csDMARDs (409)	205	24

Abbreviations: csDMARDs, conventional synthetic disease-modifying antirheumatic drugs; PRISMA, Preferred Reporting Items for Systematic Reviews and Meta-analyses; TNF, tumor necrosis factor.

**Figure 2.**
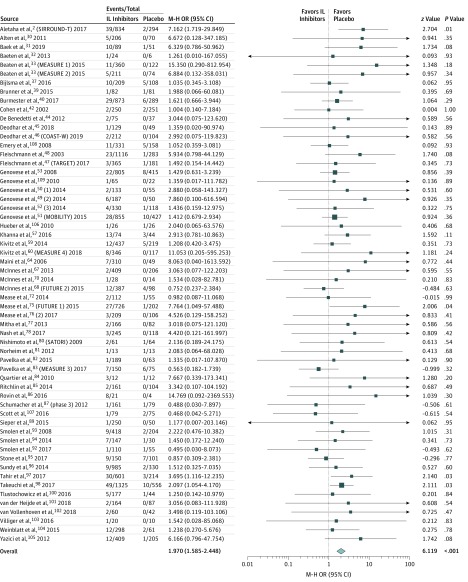
Risk of Serious Infections Size of box indicates relative weights of the studies. IL indicates interleukin; M-H, Mantel-Haenszel; and OR, odds ratio.

Sixty-nine studies included data for serious infections across all rheumatic diseases. The median duration of the trials and/or safety follow-up was 24 weeks (range, 4-156 weeks) (eTable 1 in the Supplement). A total of 24 236 patients were included in the analysis; of these, 17 177 were assessed in the treatment arms and 7059 were evaluated in the placebo arms. There were 486 events in the treatment arms and 96 events in the placebo arms. In pooled analyses, patients receiving IL inhibitors had a higher risk of serious infections vs placebo (OR, 1.97; 95% CI, 1.58-2.44; *P* < .001; *I*^2^ = 0%; high certainty) (Figure 2). The results of sensitivity analysis without continuity correction were similar (OR, 1.93; 95% CI, 1.56-2.39; *P* < .001; *I*^2^ = 0%) (eFigure 1 in the Supplement). Cumulative meta-analysis showed that the overall OR did not change after 19 studies (eFigure 2 in the Supplement). The subgroup analyses for individual medications and each disease are summarized and displayed with forest plots in eFigures 3-6 in the Supplement.

A total of 14 trials reported the incidence of opportunistic infections. The median duration of trial and/or safety follow-up was 54 weeks (range, 24-264 weeks). These trials included 9998 patients (7153 in the treatment groups; 2845 patients in the placebo groups) (eTable 2 in the Supplement). There were 43 events in the treatment groups and 5 events in the placebo groups. The following opportunistic infections were reported: 23 oral candidiasis, 9 herpes zoster, 4 esophageal candidiasis, 1 unspecified candidiasis, 2 *Mycobacterium tuberculosis*, 2 atypical mycobacterial infections, 1 histoplasmosis, and 6 unspecified.

The pooled analysis showed an increased risk of opportunistic infections with the use of IL inhibitors compared with placebo (OR, 2.35; 95% CI, 1.09-5.05; *P* = .03; *I*^2^ = 0%; moderate certainty) (eFigure 7 in the Supplement). The results of sensitivity analysis without continuity correction were not statistically significant (OR, 1.95; 95% CI, 0.99-3.82; *P* = .05; *I*^2^ = 0%) (eFigure 8 in the Supplement). Cumulative meta-analysis showed that overall OR did not change after 6 studies (eFigure 9 in the Supplement).

**Figure 3.**
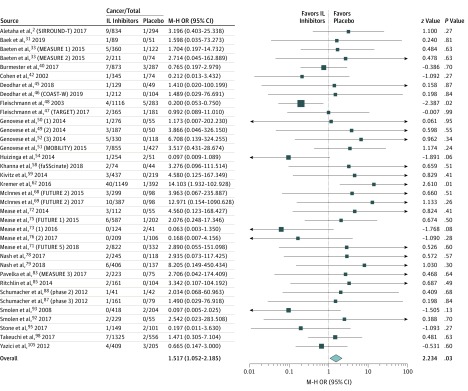
Risk of Cancer Size of box indicates relative weights of the studies. IL indicates interleukin; M-H, Mantel-Haenszel; and OR, odds ratio.

Forty-five studies with a total of 21 065 patients reported data on the incidence and type of cancers across all rheumatic diseases (eTable 3 in the Supplement). The median duration of trial and/or safety follow-up was 28 weeks (range, 12-264 weeks). There were 15 244 patients in the treatment arms and 5821 in the placebo arms. A total of 141 cases of cancer were reported in the treatment groups and 28 in the control groups. The pooled analysis demonstrated an increased risk for cancer with IL inhibitors vs placebo (OR, 1.52; 95% CI, 1.05-2.19; *P* = .03; *I*^2^ = 11%; moderate certainty) (Figure 3). The results of the sensitivity analysis without continuity correction were similar (OR, 1.47; 95% CI, 1.04-2.08; *P* = .03; *I*^2^ = 7%) (eFigure 10 in the Supplement). Cumulative meta-analysis showed that overall OR did not change after 21 studies (eFigure 11 in the Supplement).

We calculated the NNH for all primary outcomes. The NNH was 67 for 1 additional serious infection within a median follow-up of 24 weeks. The NNH for cancer was 250 (median follow-up, 28 weeks) and, for opportunistic infections, 250 (median follow-up, 54 weeks).

Using the fixed-effects model, we observed that duration of drug use was significantly associated with the effect size for cancer outcome (eFigure 12 in the Supplement). With each unit (weeks) increase in duration of drug use, the odds of cancer were increased (coefficient, 0.012; SE, 0.004; 95% CI, 0.005-0.019; *z* value, 3.22; *P* = .001). However, there were no significant associations of duration of drug use with serious (coefficient, 0.002; SE, 0.003; 95% CI, −0.004 to 0.009; *z* value; 0.67; *P* = .50) or opportunistic (coefficient, 0.003; SE, 0.008; 95% CI, −0.012 to 0.019; *z* value, 0.43; *P* = .66) infections (eFigure 13 and eFigure 14 in the Supplement).

The Egger regression test for small-study effect was statistically significant for serious infections (Egger intercept, 0.47; *P* = .01) but not for opportunistic infections (Egger intercept, 0.47; *P* = .27) or cancer (Egger intercept, 0.78; *P* = .07) (eFigure 15 in the Supplement). The funnel plot for serious infections appeared to be asymmetric, while the funnel plots for opportunistic infections and cancer were largely symmetric (eFigures 16-18 in the Supplement). However, imputation of an adjusted effect size using the trim-and-fill method did not show an important change in the effect size (OR, 1.34; 95% CI, 1.08-1.66) (eFigure 19 in the Supplement). This minimal change suggests that the overall certainty in the estimate of this outcome is not importantly affected by publication bias.^110^

In this meta-analysis, the certainty in evidence was rated as high for the outcome of serious infections for the following reasons: (1) the evidence was derived from randomized clinical trials, (2) the meta-analytic effect estimates were precise, (3) the results were consistent (heterogeneity was low or moderate across studies) (eFigure 20 in the Supplement), and (4) the majority of the randomized clinical trials included in our study are characterized by low or unclear risk of bias, as assessed with the Cochrane Collaboration’s tool (eFigure 21 and eFigure 22 in the Supplement). While the publication bias was suspected, the adjusted effect size using the trim-and-fill method was similar. However, the certainty rating of evidence was decreased to moderate for the outcomes of opportunistic infections and cancer owing to imprecision caused by the small number of events, which caused wide 95% CIs with lower boundaries close to the null effect. The absolute risk difference with intervention per 1000 patients compared with baseline risk (placebo) was 13 per 1000 patients for serious infections (95% CI, 8-19 more; NNH, 67), 2 per 1000 patients for opportunistic infections (95% CI, 0-7 more; NNH, 250), and 2 per 1000 patients for cancer (95% CI, 0-6 more; NNH, 250). The summary of this evidence using the GRADE approach is detailed in eTable 4 in the Supplement.

## Discussion

The pooled results from 74 randomized clinical trials (n = 29 214) suggests that the risk of serious infections, opportunistic infections, and cancer is increased in patients with rheumatologic diseases who are treated with IL inhibitors compared with placebo. This association is warranted by at least moderate certainty using the GRADE approach. The results are robust; the cumulative meta-analysis suggests that estimates are stable, and subgroup analysis based on drugs and disease state showed consistent results. Subgroups for individual drugs (ixekizumab, rilonacept, sarilumab, ustekinumab, brodalumab, and guselkumab) or diseases (ankylosing spondylitis, gout, juvenile idiopathic arthritis, and systemic lupus erythematosus) with a limited number of trials suggested that the risk of serious infections may be increased, but results were not statistically significant, likely reflecting the fewer number of events and small sample sizes.

Several smaller studies of IL inhibitors in individual rheumatic diseases have demonstrated an increased risk of infections, which is consistent with our study.^10,111,112^ There have also been several systematic reviews addressing the efficacy and safety of IL-1 inhibition in rheumatoid arthritis with similar findings.^18,113,114^ These studies assessed the infection risk of IL-1 inhibition, but we believe our study is unique in assessing infection risk across all IL inhibitors and is more comprehensive. Our findings are also comparable with the safety profile of TNF inhibitors in rheumatic diseases, suggesting an increased risk of serious infections.^14,18,115^ This finding of an increased number of serious infections is in contrast to a Cochrane database systematic review that compared the adverse effects of biologics (TNF inhibitors, IL-1 antagonist [anakinra], IL-6 antagonist [tocilizumab], anti-CD28 [abatacept], and anti–B cell [rituximab]) in patients with any disease and reported an increased risk of serious infections that was not statistically significant compared with placebo.^116^ A later meta-analysis of 106 randomized clinical trials showed that the risk of serious infections was increased in patients with rheumatoid arthritis treated with biologics compared with nonbiologic, traditional disease-modifying antirheumatic drugs, supporting the findings of this analysis.^18^

The existing evidence for risk of opportunistic infections with IL inhibitor therapy is not yet well established. However, several studies have investigated the risk of opportunistic infections with the use of TNF inhibitors. A meta-analysis involving 32 504 patients with rheumatoid arthritis found that biologic agents (abatacept, adalimumab, anakinra, certolizumab pegol, etanercept, golimumab, infliximab, rituximab, and tocilizumab) appeared to be associated with a small, but significant, risk of specific opportunistic infections (Peto OR; 1.79; 95% CI, 1.17-2.74) compared with placebo or disease-modifying antirheumatic drugs.^117^ Similarly, a French registry (RATIO) collected all cases of nontuberculosis opportunistic infections in patients receiving TNF inhibitors for any indication and reported a 10 times higher incidence of opportunistic infections compared with the general population.^118^ Another retrospective cohort study involving 236 531 patients reported that the crude incidence and risk of nonviral opportunistic infections among new users of TNF inhibitors compared with those initiating nonbiologic disease-modifying antirheumatic drugs was 2.7 vs 1.7 per 1000 person-years (adjusted hazard ratio, 1.6; 95% CI, 1.0-2.6).^119^ The increased risk of opportunistic infections demonstrated in our study may suggest that the safety profile of IL inhibitors is likely similar to that of TNF inhibitors.

To our knowledge, the safety data regarding the risk of cancer with IL inhibitor therapy have been limited to individual clinical trials in the absence of combined analysis. The findings in our study suggest that the risk of cancer may be increased with longer IL inhibitor therapy. Although this analysis indicated increased cancer risk with time, it is not conclusive. This increased safety signal should be investigated further by long-term clinical data. Meanwhile, caution must be practiced to adhere to the age-appropriate cancer screening guidelines, and annual screening for skin cancers should also be considered. The studies evaluating the use of IL inhibitors in psoriasis have suggested that risk of cancer is less than or comparable to the general population; the evidence is not sufficient to draw definite conclusions, however, and the evidence may not be generalizable to patients with other rheumatic diseases.^11,120,121,122,123,124,125^ Several studies have investigated the risk of cancer with TNF inhibitor therapy, but results are mixed.^14,19,126,127,128,129^ In one meta-analysis, Bongartz et al^14^ reported an increased risk for cancer (OR, 3.3; 95% CI, 1.2-9.1) with the use of anti-TNF medications (infliximab and adalimumab). However, several studies suggested no increased risk of overall cancers with TNF inhibitors.^19,126,127,128,129^

### Strengths and Limitations

Our study has several strengths. The analysis is comprehensive, and the results are robust and consistent across subgroups. We have adjusted for publication bias, and we provided not only the assessment for risk of bias but also evaluated the certainty of evidence using the GRADE approach. The study also has limitations. These results must be interpreted with caution because of factors intrinsic to the analysis of study-level data. This analysis assumes that the risk of infections or cancer is constant throughout the duration of treatment. Previous data have suggested that the risk for infection with TNF inhibitors is highest within the first 90 days of therapy,^130^ but to our knowledge, no data are currently available concerning use of IL inhibitors. Moreover, while not accounted for in this analysis, many patients receive other immunosuppressive medications, such as prednisone, in addition to IL inhibitors in clinical practice, which increases the risk of infections and cancers. Similarly, the short duration of follow-up in studies included in this review may not be sufficient to detect the actual cancer risk, which can take years to develop. A more applicable approach would be to assess longer-term data to evaluate cancer risk and length of IL inhibitor therapy, but this protocol was not possible through our approach of using clinical trial data. Furthermore, several clinical trials included in our study had shorter durations of follow-up in the placebo groups compared with the treatment groups. We included the longest available event data for treatment groups that could have biased the results to an overestimation of the true risk, as there was longer follow-up in the treatment groups to detect an adverse event compared with the placebo groups. In addition, we did not consider the differential risk associated with low or high dosages, which may underestimate or overestimate the risk of adverse reactions.

## Conclusions

This systematic review and meta-analysis suggests an increased risk of serious and opportunistic infections with IL inhibitor therapy that may be comparable to those reported for other biologics approved for the treatment of rheumatic diseases. The finding of a possibly increased risk of cancer with long-term IL inhibitor treatment should be taken into consideration and needs to be confirmed by real-world data, such as long-term epidemiologic studies from registries. This analysis provides estimates of toxic effects for infections and cancer associated with the use of IL inhibitors that can inform shared decision-making when patients and clinicians are contemplating the use of IL inhibitors for rheumatologic diseases. As a future study, the comparative safety analysis among individual IL inhibitors should be considered.

## Notes

Supplement. **eFigure 1.** Sensitivity Analysis Without Continuity Correction for the Risk of Serious Infections **eFigure 2.** Cumulative Meta-analysis Assessing Risk of Serious Infections **eFigure 3.** Odds Ratios for Serious Infections Stratified by Individual Drug **eFigure 4.** Odds Ratios for Risk of Serious Infections Stratified by Individual Disease **eFigure 5.** Odds Ratios for Risk of Serious Infections Stratified by Individual Drug **eFigure 6.** Odds Ratios for Risk of Serious Infections Stratified by Individual Disease **eFigure 7.** Odds Ratios for Risk of Opportunistic Infections **eFigure 8.** Sensitivity Analysis Without Continuity Correction for the Risk of Opportunistic Infections **eFigure 9.** Cumulative Meta-analysis Assessing Risk of Opportunistic Infections **eFigure 10.** Sensitivity Analysis Without Continuity Correction for the Risk of Malignancy **eFigure 11.** Cumulative Meta-analysis Assessing Risk of Malignancy **eFigure 12.** Meta-Regression Analysis for Malignancy Outcome **eFigure 13.** Meta-Regression Analysis for Serious Infections Outcome **eFigure 14.** Meta-Regression Analysis for Opportunistic Infections Outcome **eFigure 15.** Egger’s Regression Test **eFigure 16.** Funnel Plot Assessing Symmetry and Risk of Publication Bias for Serious Infections **eFigure 17.** Funnel Plot Assessing Symmetry and Risk of Publication Bias for Opportunistic Infections **eFigure 18.** Funnel Plot Assessing Symmetry and Risk of Publication Bias for Malignancy **eFigure 19.** Adjusted Effect Size Using Trim and Fill Method for Serious Infection Outcome **eFigure 20.** Assessment of Heterogeneity **eFigure 21.** Risk of Bias Summary of the Included Studies **eFigure 22.** Risk of Bias Graph of the Included Studies **eTable 1.** Studies Included in Assessment of Risk of Serious Infections **eTable 2.** Studies Included in Assessment of Risk of Opportunistic Infections **eTable 3.** Studies Included in Assessment of Risk of Malignancy **eTable 4.** Grading of Recommendations Assessments, Development and Evaluation (GRADE) Assessment of the Strength of Evidence **eAppendix.** Specific Search Strategy
