# Error in Figure 2, Figure 3, and Results

**DOI:** 10.1001/jamanetworkopen.2020.5507

**Published:** 2020-04-10

In the Original Investigation titled “Risk of Infections and Cancer in Patients With Rheumatologic Diseases Receiving Interleukin Inhibitors: A Systematic Review and Meta-analysis”^1^ published October 18, 2019, there was an error in Figure 2. The name given twice as “Beaten” should have been “Baeten.” There was an error in Figure 3 and eFigure 10 and corresponding data given in the Results section of the abstract and in the Results section of the text.

The odds ratio for cancer should be 1.49 with lower bound of 95% CI 1.04 and upper bound of 95% CI 2.16 in the Results section of the abstract and in the Results section of the text as per corrected Figure 3 and eFigure 10. This article has been corrected.^1^
