# Supplementary material for: Risk of Infections and Cancer in Patients With Rheumatologic Diseases Receiving Interleukin Inhibitors: A Systematic Review and Meta-analysis
Source: JAMA Netw Open. 2019 Oct 18;2(10):e1913102. doi: 10.1001/jamanetworkopen.2019.13102 (PMC6813598; doi:10.1001/jamanetworkopen.2019.13102)
Supplement: Supplement. — eFigure 1. Sensitivity Analysis Without Continuity Correction for the Risk of Serious Infections eFigure 2. Cumulative Meta-analysis Assessing Risk of Serious Infections eFigure 3. Odds Ratios for Serious Infections Stratified by Individual Drug eFigure 4. Odds Ratios for Risk of Serious Infections Stratified by Individual Disease eFigure 5. Odds Ratios for Risk of Serious Infections Stratified by Individual Drug eFigure 6. Odds Ratios for Risk of Serious Infections Stratified by Individual Disease eFigure 7. Odds Ratios for Risk of Opportunistic Infections eFigure 8. Sensitivity Analysis Without Continuity Correction for the Risk of Opportunistic Infections eFigure 9. Cumulative Meta-analysis Assessing Risk of Opportunistic Infections eFigure 10. Sensitivity Analysis Without Continuity Correction for the Risk of Malignancy eFigure 11. Cumulative Meta-analysis Assessing Risk of Malignancy eFigure 12. Meta-Regression Analysis for Malignancy Outcome eFigure 13. Meta-Regression Analysis for Serious Infections Outcome eFigure 14. Meta-Regression Analysis for Opportunistic Infections Outcome eFigure 15. Egger’s Regression Test eFigure 16. Funnel Plot Assessing Symmetry and Risk of Publication Bias for Serious Infections eFigure 17. Funnel Plot Assessing Symmetry and Risk of Publication Bias for Opportunistic Infections eFigure 18. Funnel Plot Assessing Symmetry and Risk of Publication Bias for Malignancy eFigure 19. Adjusted Effect Size Using Trim and Fill Method for Serious Infection Outcome eFigure 20. Assessment of Heterogeneity eFigure 21. Risk of Bias Summary of the Included Studies eFigure 22. Risk of Bias Graph of the Included Studies eTable 1. Studies Included in Assessment of Risk of Serious Infections eTable 2. Studies Included in Assessment of Risk of Opportunistic Infections eTable 3. Studies Included in Assessment of Risk of Malignancy eTable 4. Grading of Recommendations Assessments, Development and Evaluation (GRADE) Assessment of the Strength of Evidence [file jamanetwopen-2-e1913102-s001.pdf]

Supplementary Online Content

Bilal J, Berlinberg A, Riaz IB, et al. Risk of infections and cancer in patients with rheumatologic diseases receiving interleukin inhibitors: a systematic review and meta-analysis. *JAMA Netw Open*. 2019;2(10):e1913102. doi:10.1001/jamanetworkopen.2019.13102

eFigure 1. Sensitivity Analysis Without Continuity Correction for the Risk of Serious Infections

eFigure 2. Cumulative Meta-analysis Assessing Risk of Serious Infections

eFigure 3. Odds Ratios for Serious Infections Stratified by Individual Drug

eFigure 4. Odds Ratios for Risk of Serious Infections Stratified by Individual Disease

eFigure 5. Odds Ratios for Risk of Serious Infections Stratified by Individual Drug

eFigure 6. Odds Ratios for Risk of Serious Infections Stratified by Individual Disease

eFigure 7. Odds Ratios for Risk of Opportunistic Infections

eFigure 8. Sensitivity Analysis Without Continuity Correction for the Risk of Opportunistic Infections

eFigure 9. Cumulative Meta-analysis Assessing Risk of Opportunistic Infections

eFigure 10. Sensitivity Analysis Without Continuity Correction for the Risk of Malignancy

eFigure 11. Cumulative Meta-analysis Assessing Risk of Malignancy

eFigure 12. Meta-Regression Analysis for Malignancy Outcome

eFigure 13. Meta-Regression Analysis for Serious Infections Outcome

eFigure 14. Meta-Regression Analysis for Opportunistic Infections Outcome

eFigure 15. Egger's Regression Test

eFigure 16. Funnel Plot Assessing Symmetry and Risk of Publication Bias for Serious Infections

eFigure 17. Funnel Plot Assessing Symmetry and Risk of Publication Bias for Opportunistic Infections

eFigure 18. Funnel Plot Assessing Symmetry and Risk of Publication Bias for Malignancy

eFigure 19. Adjusted Effect Size Using Trim and Fill Method for Serious Infection Outcome

eFigure 20. Assessment of Heterogeneity

eFigure 21. Risk of Bias Summary of the Included Studies

eFigure 22. Risk of Bias Graph of the Included Studies

eTable 1. Studies Included in Assessment of Risk of Serious Infections

eTable 2. Studies Included in Assessment of Risk of Opportunistic Infections

eTable 3. Studies Included in Assessment of Risk of Malignancy

eTable 4. Grading of Recommendations Assessments, Development and Evaluation (GRADE) Assessment of the Strength of Evidence

eAppendix. Specific Search Strategy

This supplementary material has been provided by the authors to give readers additional information about their work.

© 2019 Bilal J et al. *JAMA Network Open.*

----

eFigure 1: Sensitivity analysis without continuity correction for the risk of serious infections

Study name	MH odds ratio	Lower limit	Upper limit	Z-Value	p-Value
	Statistics for each study				
Aletaha et al. (SIRROUND-T) 2017	7.162	1.719	29.849	2.704	0.007
Alten et al. 2014	3.849	0.210	70.489	0.908	0.364
Baek et al. 2017	6.329	0.786	50.962	1.734	0.083
Baeten et al. 2013	0.830	0.030	22.869	-0.110	0.912
Baeten et al. (1) 2015	8.062	0.472	137.826	1.441	0.150
Beaten et al. (2) 2015	3.969	0.217	72.642	0.929	0.353
Bijlsma et al. 2016	1.035	0.345	3.108	0.062	0.951
Brunner et al. 2015	3.000	0.120	74.732	0.670	0.503
Burmester et al. 2017	1.621	0.666	3.944	1.064	0.287
Cohen et al. 2004	1.004	0.140	7.184	0.004	0.997
De Benedetti et al. 2012	2.551	0.119	54.501	0.599	0.549
Deodhar et al. 2018	1.156	0.046	28.847	0.088	0.930
Deodhar et al. (COAST-W) 2018	2.482	0.118	52.172	0.585	0.558
Emery et al. 2008	1.052	0.359	3.081	0.092	0.927
Fleischmann et al. 2003	5.934	0.798	44.129	1.740	0.082
Fleischmann et al. (TARGET) 2017	1.492	0.154	14.442	0.345	0.730
Genovese et al. 2008	1.429	0.631	3.239	0.856	0.392
Genovese et al. 2010	1.047	0.041	26.625	0.028	0.978
Genovese et al. (1) 2014	2.110	0.100	44.672	0.480	0.632
Genovese et al. (2) 2014	3.617	0.200	65.298	0.871	0.384
Genovese et al. (3) 2014	1.436	0.159	12.975	0.322	0.748
Genovese et al. (MOBILITY) 2015	1.412	0.679	2.934	0.924	0.355
Hueber et al. 2010	3.118	0.121	80.121	0.686	0.492
Khanna et al. 2016	2.913	0.781	10.863	1.592	0.111
Kivitz et al. 2014	1.208	0.420	3.475	0.351	0.725
Kivitz et al. (MEASURE 4) 2018	5.901	0.338	103.028	1.217	0.224
Maini et al. 2006	2.446	0.138	43.514	0.609	0.542
McInnes et al. 2013	2.534	0.121	53.020	0.599	0.549
McInnes et al. 2014	1.582	0.061	41.338	0.275	0.783
Mcinnes et al. (FUTURE 2) 2017/2015	0.752	0.237	2.384	-0.484	0.628
Mease et al. 2014	0.982	0.087	11.068	-0.015	0.988
Mease et al. (FUTURE 1) 2015	7.764	1.049	57.488	2.006	0.045
Mease et al. (2) 2016	3.610	0.185	70.538	0.846	0.397
Mitha et al. 2013	2.508	0.119	52.835	0.591	0.554
Nash et al. 2017	3.421	0.175	66.762	0.811	0.417
Nishimoto et al. (SATORI) 2009	2.136	0.189	24.175	0.613	0.540
Norheim et al. 2012	3.240	0.120	87.125	0.700	0.484
Pavelka et al. 2015	1.011	0.041	25.123	0.006	0.995
Pavelka et al. (MEASURE 3) 2017	0.563	0.182	1.739	-0.999	0.318
Quartier et al. 2010	9.211	0.423	200.594	1.412	0.158
Ritchlin et al. 2014	3.276	0.156	68.920	0.763	0.445
Rovin et al. 2016	5.667	0.270	119.077	1.116	0.264
Schumacher et al. (phase III) 2012	0.488	0.030	7.897	-0.506	0.613
Scott et al. 2016	0.468	0.042	5.271	-0.615	0.539
Sieper et al. 2015	0.607	0.024	15.120	-0.304	0.761
Smolen et al. 2008	2.222	0.476	10.382	1.015	0.310
Smolen et al. 2014	1.450	0.172	12.240	0.341	0.733
Smolen et al. 2017	0.495	0.030	8.073	-0.493	0.622
Stone et al. 2017	0.857	0.309	2.381	-0.296	0.767
Sundy et al. 2014	1.512	0.325	7.035	0.527	0.598
Tahir et al. 2017	3.695	1.116	12.235	2.140	0.032
Takeuchi et al. 2017	2.097	1.054	4.170	2.111	0.035
Tlustochowicz et al. 2016	1.250	0.142	10.979	0.201	0.840
Van der Heijde et al. 2018	2.692	0.128	56.705	0.637	0.524
van Vollenhoven et al. 2018	3.632	0.170	77.623	0.826	0.409
Villiger et al. 2016	1.615	0.060	43.247	0.286	0.775
Weinblatt et al. 2015	1.238	0.270	5.676	0.275	0.784
Yazici et al. 2011	6.166	0.796	47.754	1.742	0.082
	1.935	1.565	2.392	6.101	0.000

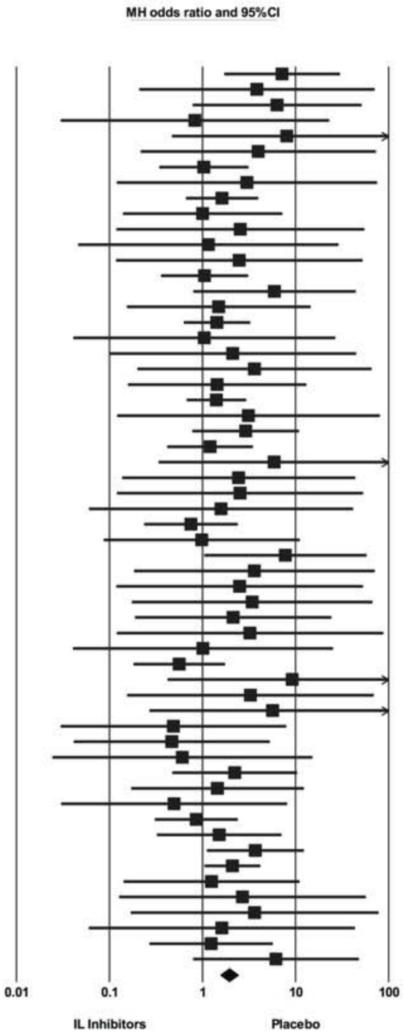

© 2019 Bilal J et al. *JAMA Network Open.*

eFigure 2: Cumulative meta-analysis assessing risk of serious infections

Study name	Point	Lower limit	Upper limit	Z-Value	p-Value
	Cumulative statistics				
Aletaha et al. (SIRROUND-T) 2017	7.162	1.719	29.849	2.704	0.007
Alten et al. 2014	7.104	1.856	27.198	2.863	0.004
Baek et al. 2017	6.902	2.225	21.413	3.344	0.001
Baeten et al. 2013	6.529	2.176	19.587	3.347	0.001
Baeten et al. (1) 2015	7.168	2.488	20.652	3.648	0.000
Beaten et al. (2) 2015	7.148	2.572	19.867	3.771	0.000
Bijlsma et al. 2016	3.854	1.901	7.812	3.742	0.000
Brunner et al. 2015	3.777	1.893	7.539	3.769	0.000
Burmester et al. 2017	2.876	1.673	4.945	3.822	0.000
Cohen et al. 2004	2.714	1.617	4.554	3.781	0.000
De Benedetti et al. 2012	2.720	1.629	4.541	3.826	0.000
Deodhar et al. 2018	2.697	1.622	4.485	3.823	0.000
Deodhar et al. (COAST-W) 2018	2.702	1.633	4.473	3.867	0.000
Emery et al. 2008	2.350	1.496	3.691	3.708	0.000
Fleischmann et al. 2003	2.524	1.627	3.916	4.130	0.000
Fleischmann et al. (TARGET) 2017	2.483	1.614	3.821	4.136	0.000
Genovese et al. 2008	2.236	1.530	3.268	4.155	0.000
Genovese et al. 2010	2.229	1.527	3.253	4.154	0.000
Genovese et al. (1) 2014	2.235	1.534	3.256	4.188	0.000
Genovese et al. (2) 2014	2.275	1.564	3.309	4.300	0.000
Genovese et al. (3) 2014	2.249	1.554	3.253	4.301	0.000
Genovese et al. (MOBILITY) 2015	2.066	1.488	2.870	4.330	0.000
Hueber et al. 2010	2.066	1.490	2.866	4.349	0.000
Khanna et al. 2016	2.108	1.534	2.896	4.603	0.000
Kivitz et al. 2014	2.024	1.494	2.741	4.554	0.000
Kivitz et al. (MEASURE 4) 2018	2.071	1.531	2.802	4.724	0.000
Maini et al. 2006	2.091	1.547	2.827	4.798	0.000
McInnes et al. 2013	2.097	1.553	2.832	4.833	0.000
McInnes et al. 2014	2.094	1.552	2.825	4.837	0.000
Mcinnes et al. (FUTURE 2) 2017/2015	1.986	1.487	2.653	4.650	0.000
Mease et al. 2014	1.969	1.478	2.624	4.627	0.000
Mease et al. (FUTURE 1) 2015	2.079	1.566	2.759	5.065	0.000
Mease et al. (2) 2016	2.092	1.578	2.774	5.127	0.000
Mitha et al. 2013	2.097	1.583	2.779	5.159	0.000
Nash et al. 2017	2.110	1.594	2.793	5.218	0.000
Nishimoto et al. (SATORI) 2009	2.110	1.597	2.788	5.253	0.000
Norheim et al. 2012	2.110	1.598	2.786	5.269	0.000
Pavelka et al. 2015	2.107	1.597	2.779	5.268	0.000
Pavelka et al. (MEASURE 3) 2017	1.977	1.514	2.581	5.005	0.000
Quartier et al. 2010	2.000	1.533	2.609	5.112	0.000
Ritchlin et al. 2014	2.007	1.540	2.616	5.153	0.000
Rovin et al. 2016	2.030	1.559	2.644	5.251	0.000
Schumacher et al. (phase III) 2012	2.008	1.544	2.611	5.201	0.000
Scott et al. 2016	1.975	1.523	2.562	5.128	0.000
Sieper et al. 2015	1.973	1.521	2.558	5.125	0.000
Smolen et al. 2008	1.979	1.532	2.557	5.223	0.000
Smolen et al. 2014	1.971	1.528	2.542	5.228	0.000
Smolen et al. 2017	1.952	1.515	2.513	5.181	0.000
Stone et al. 2017	1.872	1.466	2.391	5.030	0.000
Sundy et al. 2014	1.863	1.463	2.371	5.051	0.000
Tahir et al. 2017	1.929	1.523	2.443	5.453	0.000
Takeuchi et al. 2017	1.947	1.557	2.434	5.845	0.000
Tlustochowicz et al. 2016	1.939	1.552	2.421	5.839	0.000
Van der Heijde et al. 2018	1.942	1.556	2.425	5.867	0.000
van Vollenhoven et al. 2018	1.948	1.561	2.430	5.905	0.000
Villiger et al. 2016	1.947	1.561	2.428	5.909	0.000
Weinblatt et al. 2015	1.930	1.551	2.402	5.893	0.000
Yazici et al. 2011	1.970	1.585	2.448	6.119	0.000
	1.970	1.585	2.448	6.119	0.000

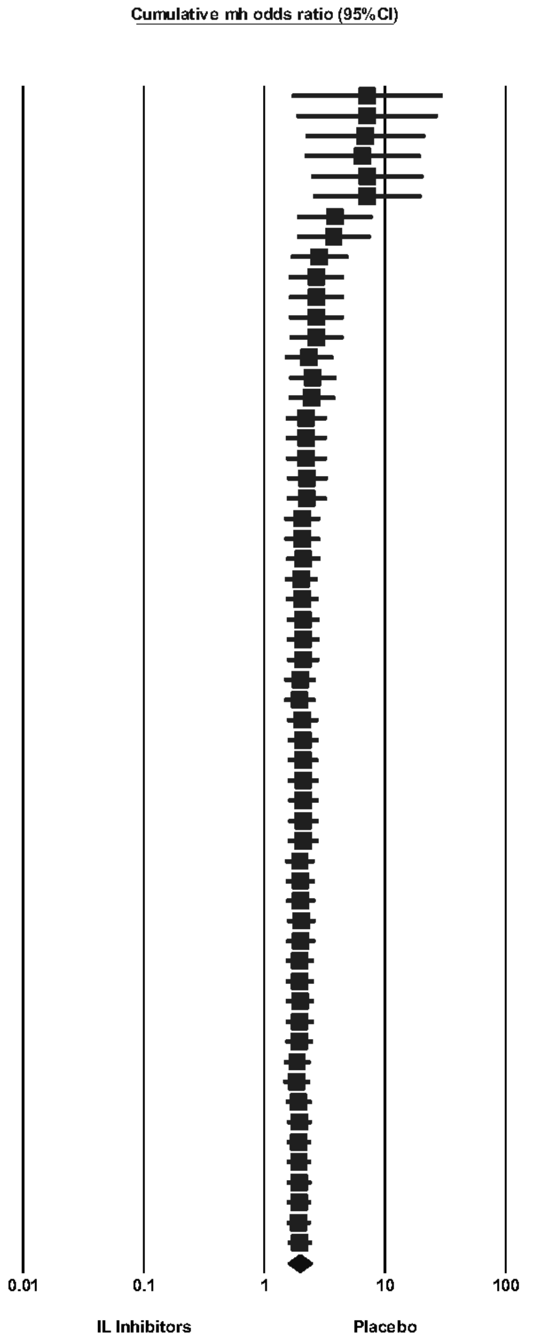

© 2019 Bilal J et al. *JAMA Network Open.*

eFigure 3: Odds ratios for serious infections stratified by individual drug
A) tocilizumab B) secukinumab C) anakinra D) Ixekizumab E) rilanocept F) sarilumab
G) sarukumab H) ustekinumab I) brodalumab J) guselkumab

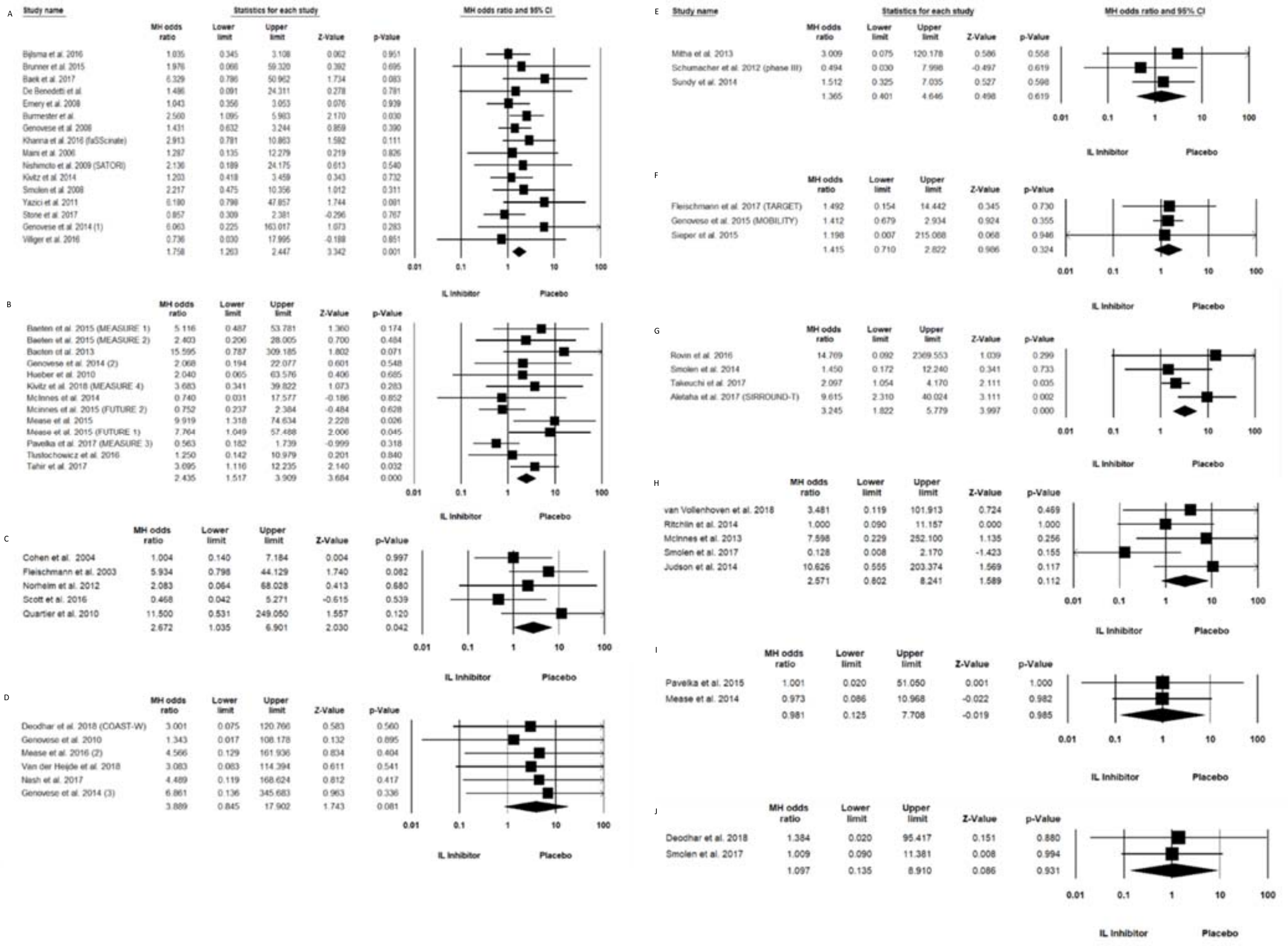

© 2019 Bilal J et al. *JAMA Network Open.*

eFigure 4: Odds ratios for risk of serious infections stratified by individual disease
A) Parthritis B) Ankylosing spondylitis C) Systemic lupus erythematosus
D) Juvenile rheumatoid arthritis E) Gout F) Rheumatoid arthritis

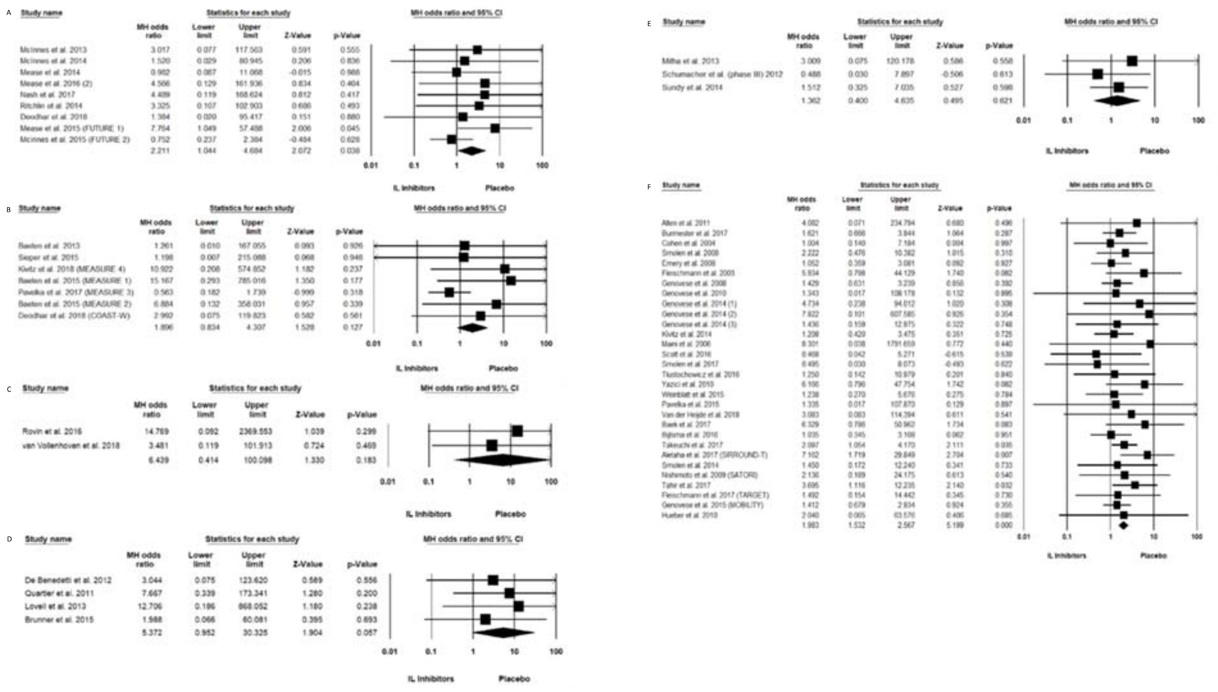

© 2019 Bilal J et al. *JAMA Network Open.*

eFigure 5: Odds ratios for risk of serious infections stratified by individual drug

Medication	OR	95% CI	p-value
**Anakinra**	2.67	1.03-6.90	0.04
**Brodalumab**	0.98	0.12-7.71	0.98
**Guselkumab**	1.09	0.13-8.91	0.93
**Ixekizumab**	3.89	0.84-17.90	0.08
**Rilonacept**	1.36	0.40-4.65	0.62
**Sarilumab**	1.41	0.71-2.82	0.32
**Secukinumab**	2.43	1.52-3.91	<.001
**Sirukumab**	3.24	1.82-5.78	<.001
**Tocilizumab**	1.76	1.26-2.45	0.001
**Ustekinumab**	2.57	0.80-8.24	0.11

© 2019 Bilal J et al. *JAMA Network Open.*

eFigure 6: Odds ratios for risk of serious infections stratified by individual disease

Disease	OR	95% CI	p-value
**Ankylosing Spondylitis**	1.896	0.83-4.31	0.13
**Gout**	1.36	0.40-4.63	0.62
**Juvenile Idiopathic Arthritis**	5.37	0.95-30.32	0.06
**Psoriatic Arthritis**	2.21	1.04-4.68	0.04
**Rheumatoid Arthritis**	1.98	1.53-2.57	<.001
**Systemic Lupus Erythematosus**	6.44	0.42-100.09	0.18

© 2019 Bilal J et al. *JAMA Network Open.*

eFigure 7: Odds ratios for risk of opportunistic infections

Study name	Events / Total: IL Inhibitors	Events / Total: Placebo	MH odds ratio	Lower limit	Upper limit	Z-Value	p-Value
Beaten et al. (MEASURE 1) 2015 [33]	2 / 360	0 / 122	2.688	0.043	168.979	0.468	0.64
Baeten et al. (MEASURE 2) 2015 [33]	2 / 211	0 / 74	2.714	0.045	162.889	0.478	0.63
Burmester et al. 2017 [40]	3 / 1157	0 / 287	3.747	0.040	350.618	0.570	0.57
Deodhar et al. 2018 [45]	1 / 129	0 / 49	1.384	0.020	95.417	0.151	0.88
Fleischmann et al. (TARGET) 2017 [47]	2 / 365	1 / 181	0.992	0.089	11.010	-0.007	0.99
Genovese et al. 2008 [53]	1 / 802	0 / 415	1.518	0.031	74.144	0.210	0.83
Genovese et al. (MOBILITY) 2015 [51]	7 / 855	2 / 427	1.754	0.363	8.480	0.699	0.48
Ilowite et al. 2009 [55]	3 / 25	1 / 25	6.682	0.317	140.911	1.221	0.22
Kivitz et al. 2014 [59]	1 / 437	1 / 219	0.333	0.015	7.417	-0.695	0.49
Kremer et al. 2011 [61]	1 / 1149	0 / 392	1.343	0.017	104.761	0.133	0.89
Mcinnes et al. (FUTURE 2) 2015 [68]	11 / 387	0 / 98	14.164	0.173	1161.002	1.179	0.24
Mease et al. (2) 2017 [76]	2 / 209	0 / 106	3.038	0.078	118.286	0.595	0.55
Mease et al. (FUTURE 5) 2018 [74]	4 / 822	1 / 332	1.619	0.180	14.535	0.430	0.67
Nash et al. 2017 [78]	4 / 245	0 / 118	6.010	0.167	215.959	0.981	0.33
**Overall**			2.346	1.090	5.053	2.179	0.03

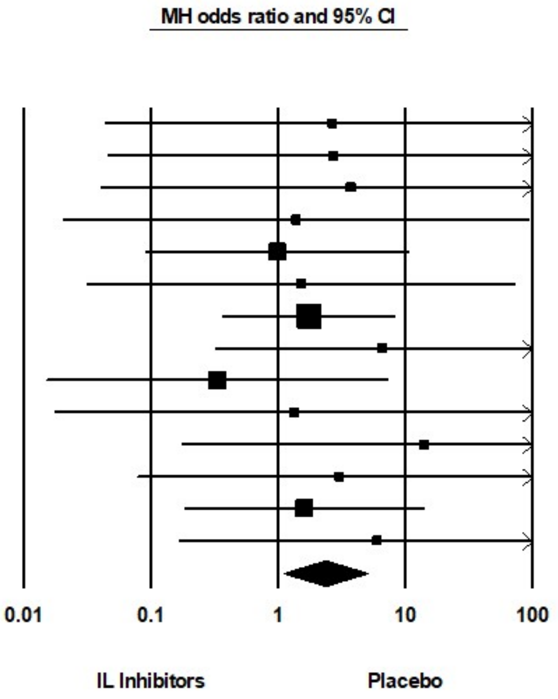

© 2019 Bilal J et al. *JAMA Network Open.*

eFigure 8: Sensitivity analysis without continuity correction for the risk of opportunistic infections

Study name	Statistics for each study				
	MH odds ratio	Lower limit	Upper limit	Z-Value	p-Value
Baeten et al. 2015 (MEASURE 1)	1.709	0.081	35.834	0.345	0.730
Baeten et al. 2015 (MEASURE 2)	1.778	0.084	37.463	0.370	0.711
Burmester et al. 2017	1.743	0.090	33.842	0.367	0.713
Deodhar et al. 2018	1.156	0.046	28.847	0.088	0.930
Fleischmann et al. 2017 (TARGET)	0.992	0.089	11.010	-0.007	0.995
Genovese et al. 2008	1.555	0.063	38.260	0.270	0.787
Genovese et al. 2015 (MOBILITY)	1.754	0.363	8.480	0.699	0.485
Ilowite et al. 2009	7.933	0.388	162.066	1.345	0.178
Kivitz et al. 2014	0.166	0.007	4.103	-1.097	0.273
Kremer et al. 2011	1.025	0.042	25.219	0.015	0.988
Mcinnes et al. 2015 (FUTURE 2)	6.017	0.352	103.000	1.238	0.216
Mease et al. 2016 (2)	2.566	0.122	53.936	0.607	0.544
Mease et al. 2018 (FUTURE 5)	1.619	0.180	14.535	0.430	0.667
Nash et al. 2017	4.416	0.236	82.704	0.994	0.320
	1.947	0.992	3.821	1.938	0.053

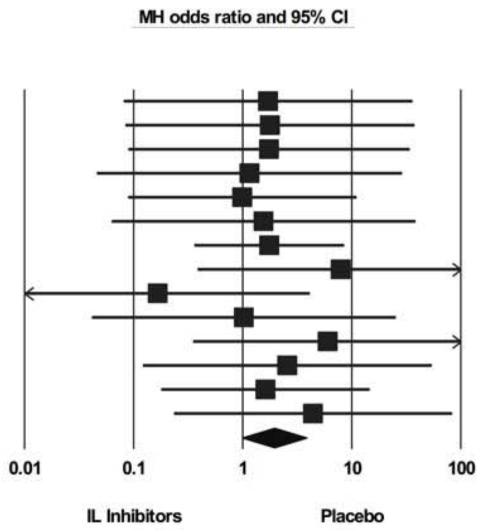

© 2019 Bilal J et al. *JAMA Network Open.*

eFigure 9: Cumulative meta-analysis assessing risk of opportunistic infections

Study name	Cumulative statistics				
	Point	Lower limit	Upper limit	Z-Value	p-Value
Baeten et al. (MEASURE 1) 2015	2.688	0.043	168.979	0.468	0.640
Baeten et al. (MEASURE 2) 2015	2.701	0.147	49.665	0.669	0.504
Burmester et al. 2017	3.011	0.260	34.830	0.882	0.378
Deodhar et al. 2018	2.573	0.314	21.107	0.880	0.379
Fleischmann et al. (TARGET) 2017	1.822	0.387	8.572	0.760	0.447
Genovese et al. 2008	1.780	0.423	7.496	0.786	0.432
Genovese et al. (MOBILITY) 2015	1.769	0.611	5.115	1.052	0.293
Ilowite et al. 2009	2.110	0.779	5.714	1.469	0.142
Kivitz et al. 2014	1.801	0.717	4.526	1.252	0.211
Kremer et al. 2011	1.780	0.723	4.384	1.254	0.210
Mcinnes et al. (FUTURE 2) 2015	2.244	0.941	5.348	1.824	0.068
Mease et al. (2) 2016	2.284	0.981	5.316	1.916	0.055
Mease et al. (FUTURE 5) 2018	2.192	0.997	4.819	1.951	0.051
Nash et al. 2017	2.346	1.090	5.053	2.179	0.029
	2.346	1.090	5.053	2.179	0.029

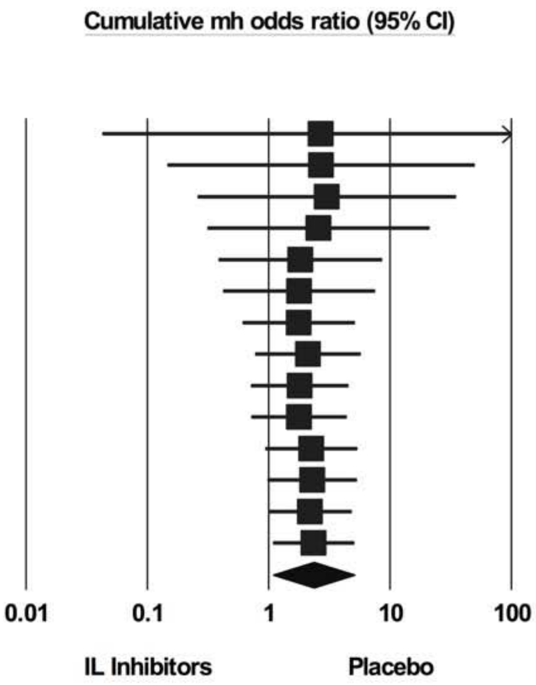

© 2019 Bilal J et al. *JAMA Network Open.*

eFigure 10: Sensitivity analysis without continuity correction for the risk of malignancy

Study name	Statistics for each study				
	MH odds ratio	Lower limit	Upper limit	Z-Value	p-Value
Aletaha et al. 2017 (SIRROUND-T)	3.196	0.403	25.338	1.100	0.271
Baek et al. 2017	1.746	0.070	43.649	0.339	0.734
Beaten et al. 2015 (1)	1.704	0.197	14.732	0.484	0.628
Beaten et al. 2015 (2)	1.778	0.084	37.463	0.370	0.711
Burmester et al. 2017	0.765	0.197	2.979	-0.386	0.700
Cohen et al. 2002	0.212	0.013	3.432	-1.092	0.275
Deodhar et al. 2018	1.156	0.046	28.847	0.088	0.930
Deodhar et al. 2018 (COAST-W)	1.482	0.060	36.699	0.240	0.810
Fleischmann et al. 2003	0.200	0.053	0.750	-2.387	0.017
Fleischmann et al. 2017 (TARGET)	0.992	0.089	11.010	-0.007	0.995
Genovese et al. 2014 (1)	0.604	0.024	15.029	-0.307	0.759
Genovese et al. 2014 (2)	1.916	0.097	37.702	0.428	0.669
Genovese et al. 2014 (3)	4.005	0.220	72.976	0.937	0.349
Genovese et al. 2015 (MOBILITY)	3.517	0.431	28.674	1.174	0.240
Huizinga et al. 2014	0.097	0.009	1.089	-1.891	0.059
Khanna et al. 2016, 2018 (faSScinate)	3.069	0.144	65.402	0.718	0.473
Kivitz et al. 2014	3.536	0.182	68.765	0.834	0.404
Kremer et al. 2011, 2016	14.103	1.932	102.928	2.610	0.009
McInnes et al. 2015	2.325	0.119	45.416	0.557	0.578
Mcinnes et al. 2017, 2015 (FUTURE 2)	5.479	0.318	94.320	1.172	0.241
Mease et al. 2014	3.548	0.180	69.903	0.833	0.405
Mease et al. 2015 (FUTURE 1)	2.076	0.248	17.346	0.674	0.500
Mease et al. 2016 (1)	0.063	0.003	1.350	-1.768	0.077
Mease et al. 2016 (2)	0.505	0.031	8.151	-0.482	0.630
Mease et al. 2018 (FUTURE 5)	2.026	0.097	42.317	0.455	0.649
Nash et al. 2017	2.433	0.116	51.087	0.573	0.567
Nash et al. 2018	4.463	0.250	79.744	1.017	0.309
Pavelka et al. 2017 (MEASURE 3)	1.704	0.081	35.900	0.343	0.732
Ritchlin et al. 2014	3.276	0.156	68.920	0.763	0.445
Schumacher et al. (phase II) 2012	3.148	0.125	79.538	0.696	0.486
Schumacher et al. (phase III) 2012	1.486	0.060	36.890	0.242	0.809
Smolen et al. 2008	0.097	0.005	2.025	-1.505	0.132
Smolen et al. 2017	1.220	0.058	25.769	0.128	0.898
Stone et al. 2017	0.133	0.006	2.802	-1.297	0.195
Takeuchi et al. 2017	1.471	0.305	7.104	0.481	0.631
Yazici et al. 2010	0.665	0.147	3.000	-0.531	0.596
	1.471	1.039	2.082	2.176	0.030

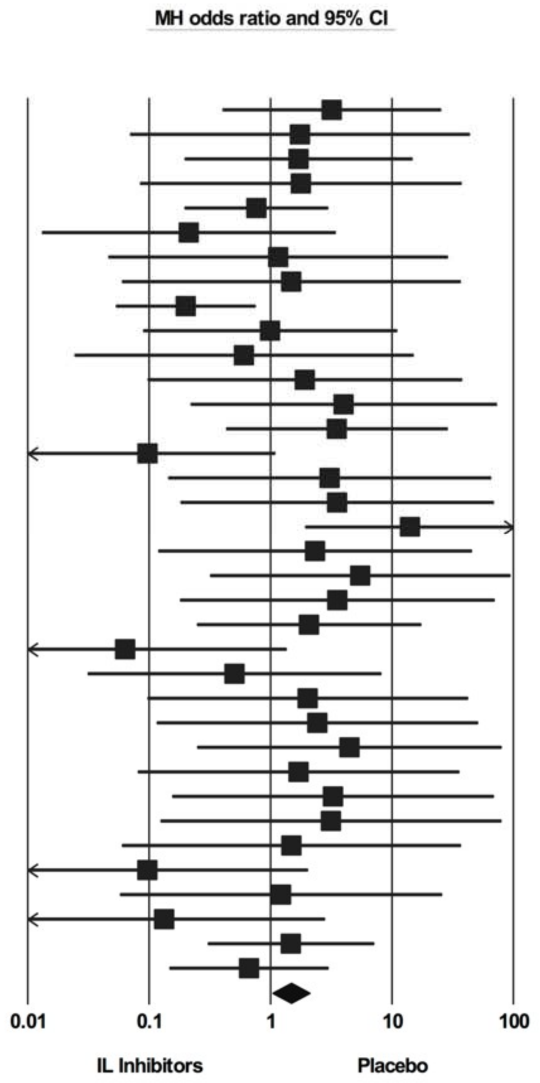

© 2019 Bilal J et al. *JAMA Network Open.*

eFigure 11: Cumulative meta-analysis assessing risk of malignancy

Study name	Cumulative statistics				
	Point	Lower limit	Upper limit	Z-Value	p-Value
Aletaha et al. (SIRROUND-T) 2017	3.196	0.403	25.338	1.100	0.271
Baek et al. 2017	2.819	0.462	17.194	1.123	0.261
Beaten et al. (1)* 2015	2.334	0.586	9.293	1.203	0.229
Beaten et al. (2)* 2015	2.373	0.641	8.784	1.294	0.196
Burmester et al. 2017	1.500	0.597	3.769	0.862	0.389
Cohen et al. 2002	1.286	0.546	3.026	0.576	0.565
Deodhar et al. 2018	1.291	0.558	2.987	0.596	0.551
Deodhar et al. (COAST-W) 2018	1.299	0.572	2.951	0.624	0.532
Fleischmann et al. 2003	0.831	0.431	1.604	-0.552	0.581
Fleischmann et al. (TARGET) 2017	0.842	0.446	1.587	-0.532	0.594
Genovese et al. (1) 2014	0.846	0.451	1.588	-0.520	0.603
Genovese et al. (2) 2014	0.894	0.481	1.661	-0.354	0.723
Genovese et al. (3) 2014	1.003	0.548	1.835	0.008	0.993
Genovese et al. (MOBILITY) 2015	1.152	0.647	2.050	0.480	0.631
Huizinga et al. 2014	1.015	0.588	1.753	0.054	0.957
Khanna et al. (faSScinate) 2018	1.054	0.615	1.807	0.192	0.848
Kivitz et al. 2014	1.112	0.654	1.891	0.393	0.695
Kremer et al. 2016	1.781	1.087	2.916	2.293	0.022
McInnes et al. 2015	1.809	1.109	2.952	2.375	0.018
McInnes et al. (FUTURE 2) 2017	1.931	1.189	3.134	2.661	0.008
Mease et al. 2014	1.970	1.219	3.183	2.768	0.006
Mease et al. (FUTURE 1) 2015	1.975	1.237	3.153	2.849	0.004
Mease et al. (1) 2016	1.767	1.131	2.759	2.502	0.012
Mease et al. (2) 2016	1.679	1.087	2.595	2.336	0.020
Mease et al. (FUTURE 5) 2018	1.693	1.098	2.608	2.385	0.017
Nash et al. 2017	1.707	1.111	2.623	2.442	0.015
Nash et al. 2018	1.771	1.157	2.712	2.630	0.009
Pavelka et al. (MEASURE 3) 2017	1.780	1.165	2.720	2.668	0.008
Ritchlin et al. 2014	1.799	1.182	2.740	2.738	0.006
Schumacher et al. (phase II) 2012	1.802	1.187	2.736	2.766	0.006
Schumacher et al. (phase III) 2012	1.799	1.188	2.724	2.773	0.006
Smolen et al. 2008	1.665	1.115	2.486	2.493	0.013
Smolen et al. 2017	1.671	1.121	2.491	2.520	0.012
Stone et al. 2017	1.594	1.079	2.354	2.342	0.019
Takeuchi et al. 2017	1.587	1.086	2.317	2.389	0.017
Yazici et al. 2010	1.517	1.052	2.185	2.234	0.025
	1.517	1.052	2.185	2.234	0.025

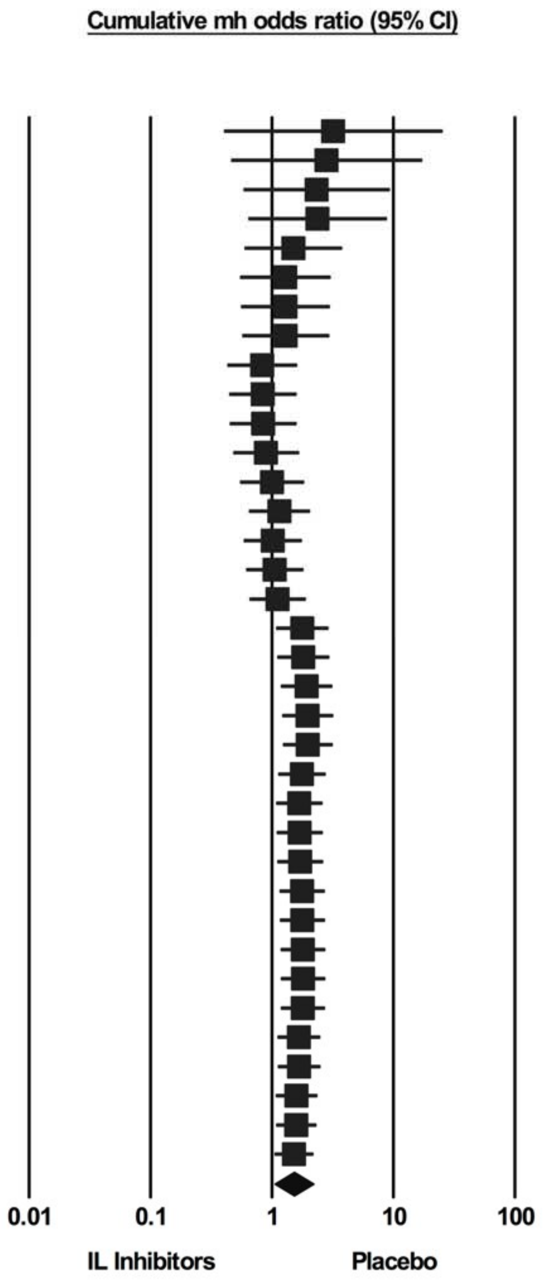

© 2019 Bilal J et al. *JAMA Network Open.*

eFigure 12: Meta-regression analysis for malignancy outcome

Covariate	Coefficient	Standard Error	95% Lower	95% Upper	Z-value	2-sided P-value
Intercept	-0.7198	0.3295	-1.3656	-0.0741	-2.18	0.0289
Time	0.0120	0.0037	0.0047	0.0194	3.22	0.0013

Analysis of variance

	Q	df	p
Model	10.3781	1.0000	0.0013
Residual	26.3952	34.0000	0.8210
Total	36.7734	35.0000	0.3867

Number of studies in the analysis **36**

© 2019 Bilal J et al. *JAMA Network Open.*

eFigure 13. Meta-regression analysis for serious infections outcome

Covariate	Coefficient	Standard Error	95% Lower	95% Upper	Z-value	2-sided P-value
**Intercept**	0.3873	0.2233	-0.0504	0.8250	1.73	0.0828
**Time**	0.0024	0.0035	-0.0046	0.0093	0.67	0.5033

Analysis of variance

	Q	df	p
**Model**	0.4480	1.0000	0.5033
**Residual**	32.9762	56.0000	0.9940
**Total**	33.4243	57.0000	0.9946

Number of studies in the analysis 58

© 2019 Bilal J et al. *JAMA Network Open.*

eFigure 14: Meta-regression analysis for opportunistic infections outcome

Covariate	Coefficient	Standard Error	95% Lower	95% Upper	Z-value	2-sided P-value
Intercept	0.4612	0.6173	-0.7486	1.6710	0.75	0.4549
Time	0.0035	0.0081	-0.0124	0.0194	0.43	0.6650

Analysis of variance

	Q	df	p
Model	0.1875	1.0000	0.6650
Residual	3.4441	12.0000	0.9915
Total	3.6315	13.0000	0.9945

Number of studies in the analysis **14**

© 2019 Bilal J et al. *JAMA Network Open.*

eFigure 15: Egger's regression test

a) serious infections, p=0.01 b) opportunistic infections, p=0.27 c) malignancy, p=0.07

A **Egger's regression intercept**

Intercept	0.46807
Standard error	0.17734
95% lower limit (2-tailed)	0.11281
95% upper limit (2-tailed)	0.82334
t-value	2.63934
df	56.00000
P-value (1-tailed)	0.00537
P-value (2-tailed)	0.01074

B **Egger's regression intercept**

Intercept	0.46917
Standard error	0.40923
95% lower limit (2-tailed)	-0.42245
95% upper limit (2-tailed)	1.36080
t-value	1.14649
df	12.00000
P-value (1-tailed)	0.13697
P-value (2-tailed)	0.27394

C **Egger's regression intercept**

Intercept	0.78282
Standard error	0.42723
95% lower limit (2-tailed)	-0.08542
95% upper limit (2-tailed)	1.65105
t-value	1.83231
df	34.00000
P-value (1-tailed)	0.03784
P-value (2-tailed)	0.07568

© 2019 Bilal J et al. *JAMA Network Open.*

eFigure 16: Funnel plot assessing symmetry and risk of publication bias for serious infections

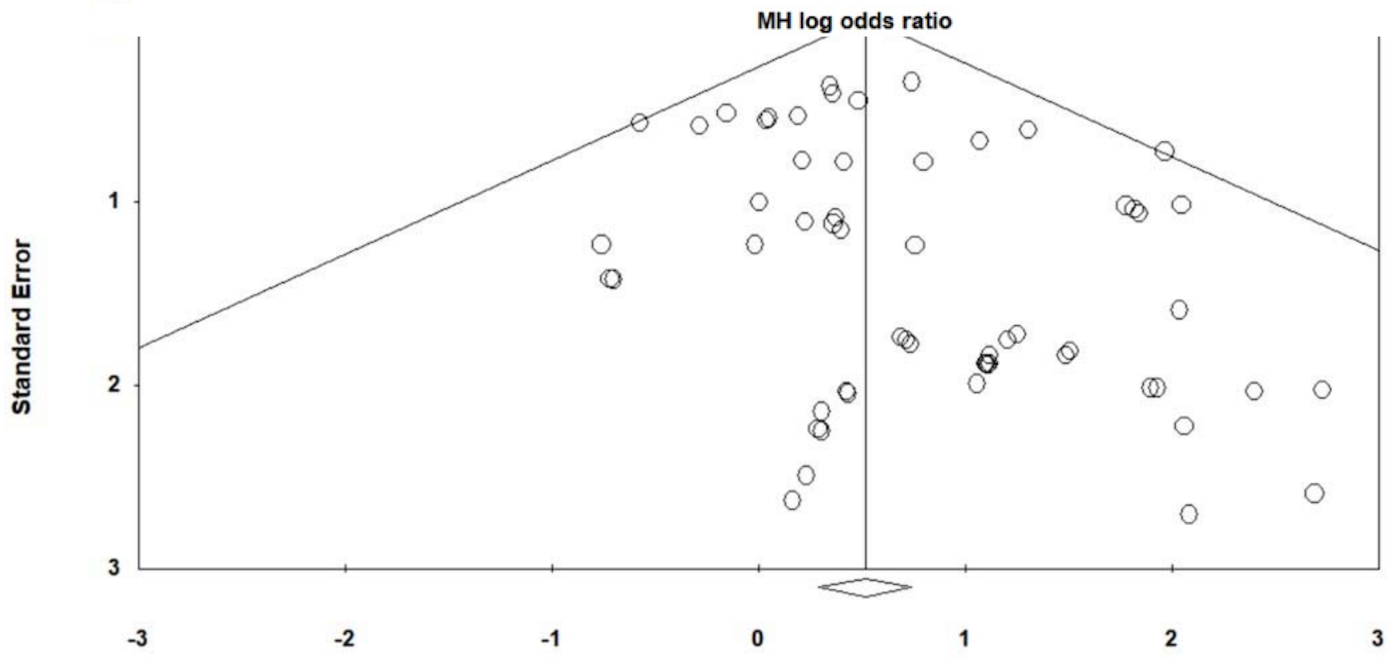

© 2019 Bilal J et al. *JAMA Network Open.*

eFigure 17: Funnel plot assessing symmetry and risk of publication bias for opportunistic infections

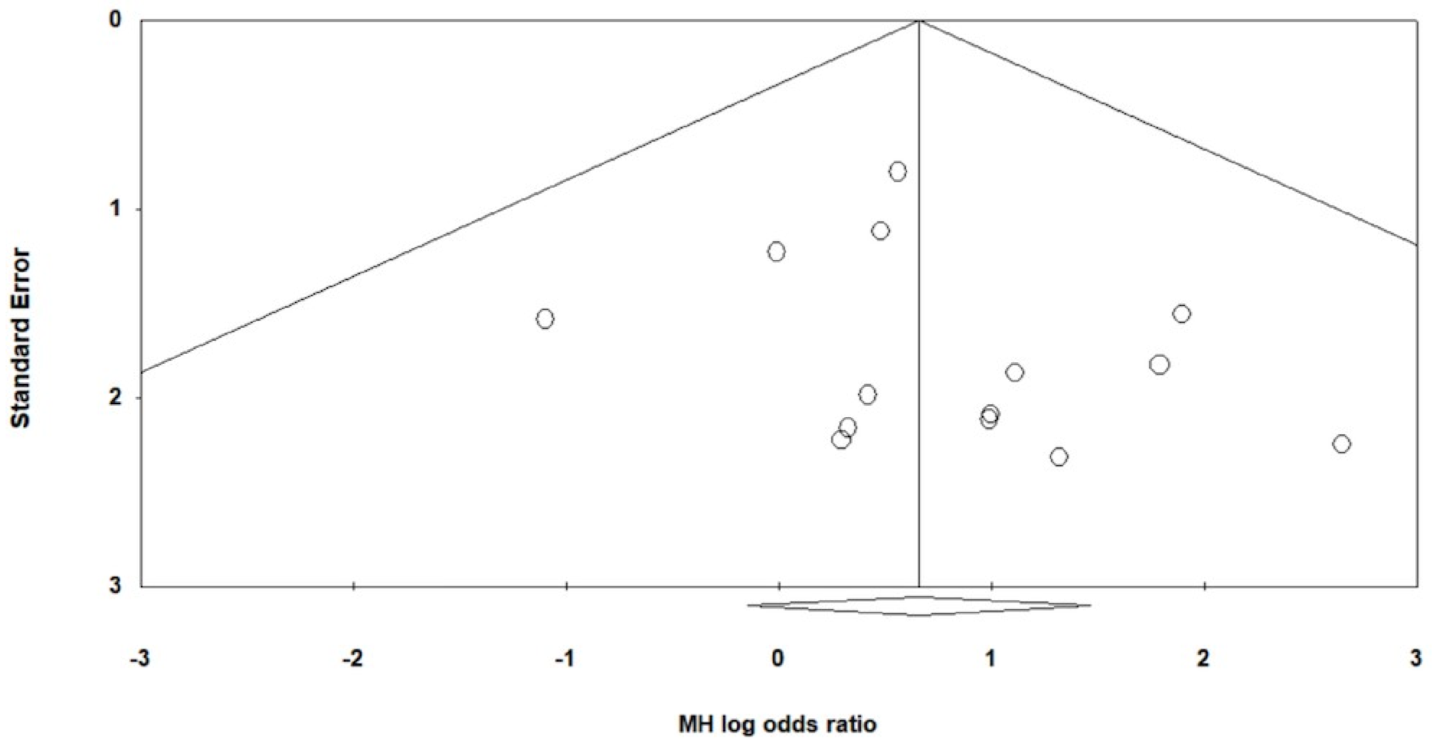

© 2019 Bilal J et al. *JAMA Network Open.*

eFigure 18: Funnel plot assessing symmetry and risk of publication bias for malignancy

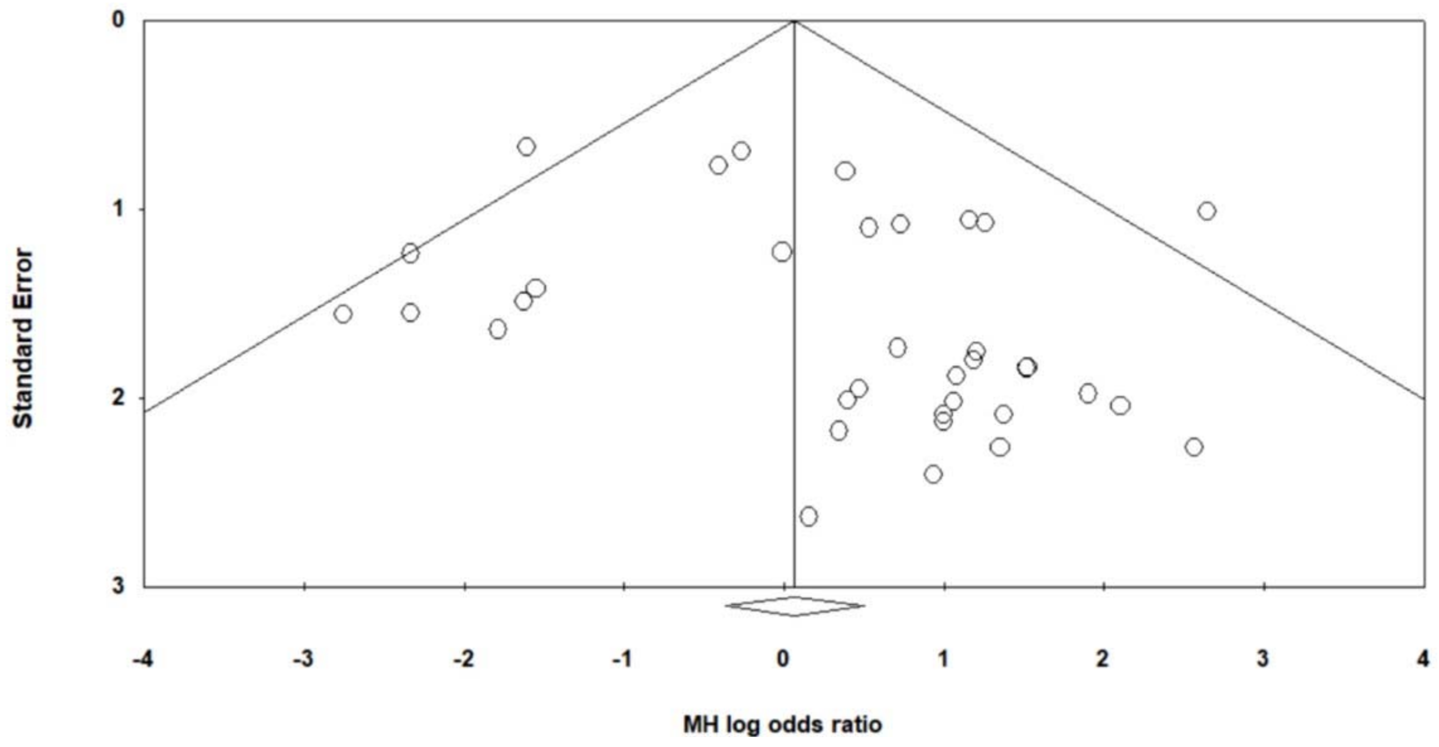

© 2019 Bilal J et al. *JAMA Network Open.*

eFigure 19: Adjusted effect size using trim and fill method for serious infection outcome

Duval and Tweedie's trim and fill

		Fixed Effects			Random Effects			Q Value
	Studies Trimmed	Point Estimate	Lower Limit	Upper Limit	Point Estimate	Lower Limit	Upper Limit	
**Observed values**		1.67418	1.33538	2.09895	1.67418	1.33538	2.09895	33.42425
**Adjusted values**	18	1.34176	1.08685	1.65646	1.34176	1.08685	1.65646	62.85502

© 2019 Bilal J et al. *JAMA Network Open.*

eFigure 20: Assessment of Heterogeneity

A) serious infections. B) opportunistic infections. C) malignancy

A

Model	Effect size and 95% interval				Test of null (2-Tail)		Heterogeneity				Tau-squared			
Model	Number Studies	Point estimate	Lower limit	Upper limit	Z-value	P-value	Q-value	df (Q)	P-value	I-squared	Tau Squared	Standard Error	Variance	Tau
Fixed	58	1.970	1.585	2.448	6.119	0.000	35.414	57	0.989	0.000	0.000	0.149	0.022	0.000
Random	58	1.674	1.335	2.099	4.467	0.000								

B

Model	Effect size and 95% interval				Test of null (2-Tail)		Heterogeneity				Tau-squared			
Model	Number Studies	Point estimate	Lower limit	Upper limit	Z-value	P-value	Q-value	df (Q)	P-value	I-squared	Tau Squared	Standard Error	Variance	Tau
Fixed	14	2.346	1.090	5.053	2.179	0.029	3.850	13	0.993	0.000	0.000	0.983	0.966	0.000
Random	14	1.936	0.865	4.334	1.606	0.108								

C

Model	Effect size and 95% interval				Test of null (2-Tail)		Heterogeneity				Tau-squared			
Model	Number Studies	Point estimate	Lower limit	Upper limit	Z-value	P-value	Q-value	df (Q)	P-value	I-squared	Tau Squared	Standard Error	Variance	Tau
Fixed	36	1.517	1.052	2.185	2.234	0.025	39.281	35	0.284	10.899	0.222	0.489	0.239	0.471
Random	36	1.126	0.698	1.818	0.487	0.626								

© 2019 Bilal J et al. *JAMA Network Open.*

eFigure 21: Risk of bias summary of the included studies (authors' judgements about each risk of bias item for each included study)
*green - low risk, yellow - unclear, red - high risk

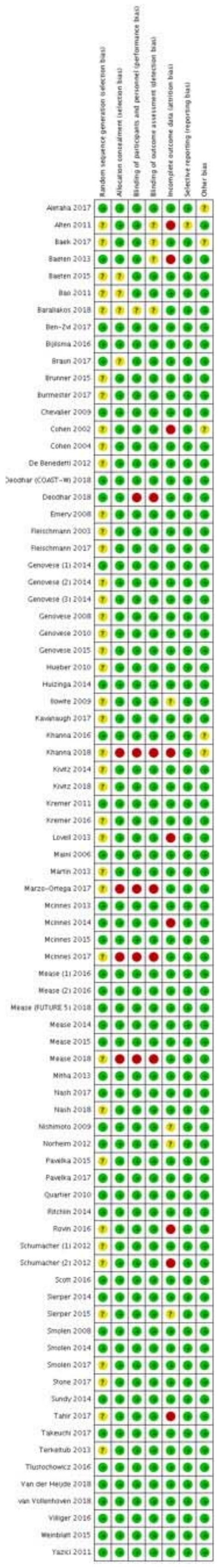

© 2019 Bilal J et al. *JAMA Network Open.*

eFigure 22: Risk of bias graph of the included studies (authors' judgements about each risk of bias item presented as percentages across all included studies)
*green - low risk, yellow - unclear, red - high risk

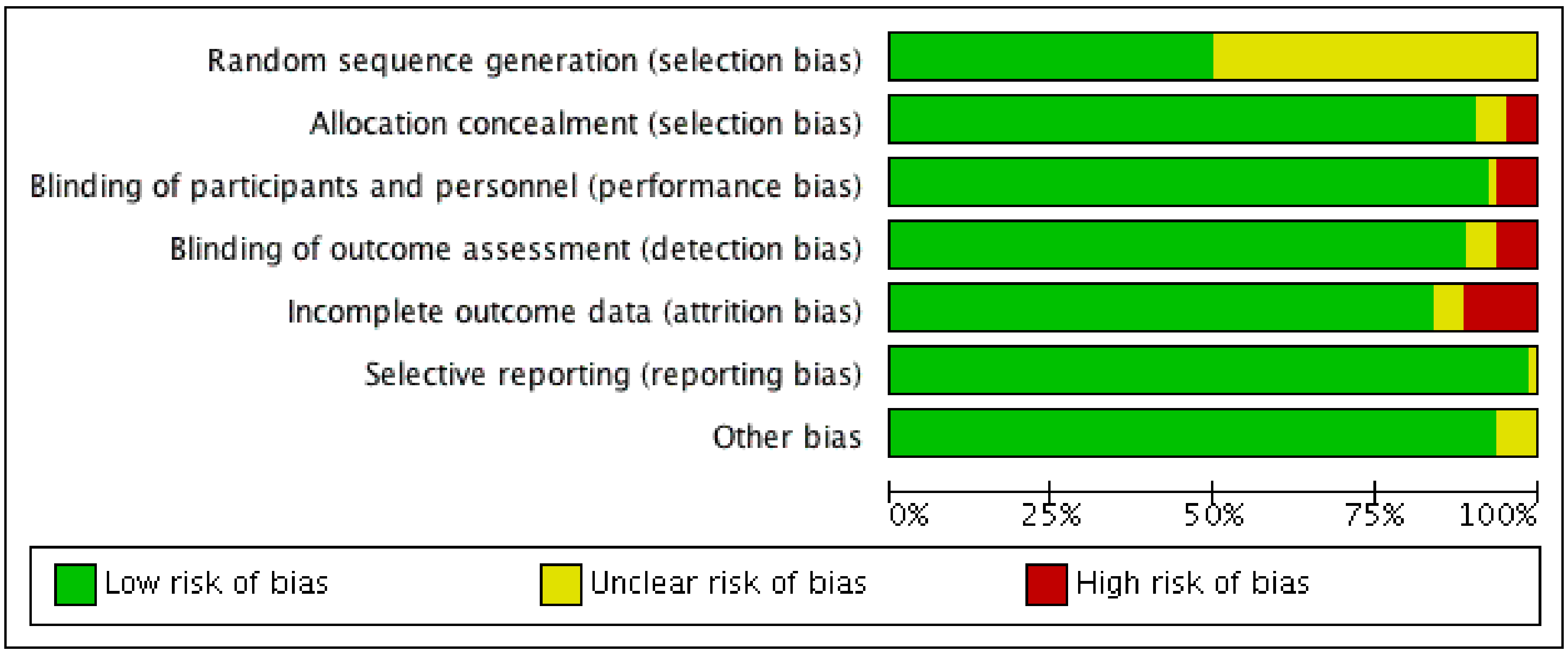

© 2019 Bilal J et al. *JAMA Network Open.*

eTable 1: Studies included in assessment of risk of serious infections

		Treatment Group		Treatment Group	
**Study**	**Drug**	**Serious Infections**	**Candida**	**Serious Infections**	**Candida**
**Aletaha et al. 2017 (SIRROUND-T)[2]**	Sirukumab	39/834	Not reported	2/294	Not reported
**Alten et al. 2011[30]**	Canakinumab	3/204	0/204	0/70	0/70
**Baek et al. 2018[31]**	Tocilizumab	10/89	Not reported	1/51	Not reported
**Baeten et al. 2015[33] Braun et al. 2017[38] Baraliakos et al. 2018**	Secukinumab	11/360	4/360	0/122	0/122
**Baeten et al. 2015[33] Marzo-Ortega et al. 2017 (MEASURE 2)[66]**	Secukinumab	5/211	4/211	0/74	0/74
**Baeten et al. 2013[32]**	Secukinumab	1/24	0/24	0/6	0/6
**Ben-Zvi et al. 2017[36]**	Anakinra	0/12	0/12	0/13	0/13
**Bijlsma et al. 2016[37]**	Tocilizumab	10/209	Not reported	5/108	Not reported
**Brunner et al. 2015[39]**	Tocilizumab	1/82	0/82	0/81	0/81
**Burmester et al. 2017[40]**	Tocilizumab	61/1107	0/1107	6/282	0/282
**Chevalier et al. 2009[41]**	Anakinra	0/101	0/101	0/69	0/69
**Cohen et al. 2004[43]**	Anakinra	2/250	0/250	2/251	0/251
**De Benedetti et al. 2012[44]**	Tocilizumab	2/75	0/75	0/37	0/37
**Deodhar et al. 2018[45]**	Guselkumab	1/129	0/129	0/49	0/49
**Deodhar et al. 2018 (COAST-W)[46]**	Ixekizumab	2/212	2/212	0/104	0/104
**Emery et al. 2008[108]**	Tocilizumab	11/338	0/338	5/160	0/160
**Fleischmann et al. 2003[48]**	Anakinra	23/1116	0/1116	1/283	0/283
**Fleischmann et al. 2017 (TARGET)[47]**	Sarilumab	3/365	Not reported	1/181	Not reported
**Genovese et al. 2008[53]**	Tocilizumab	22/802	0/802	8/414	0/414
**Genovese et al. 2010[109]**	Ixekizumab	1/65	0/65	0/22	0/22
**Genovese et al. 2014 (1)[50]**	Tocilizumab	2/43	0/43	0/55	0/55
	Olokizumab	2/133	0/133		
**Genovese et al. 2014 (2)[49]**	Secukinumab	6/187	0/187	0/50	0/50
**Genovese et al. 2014 (3)[52]**	Ixekizumab	4/330	0/330	1/118	0/118
**Genovese et al. 2015 (MOBILITY)[51]**	Sarilumab	28/855	1/855	10/427	0/427
**Hueber et al. 2010[106]**	Secukinumab	1/26	Not reported	0/26	Not reported
**Huizinga et al. 2014[54]**	Sarilumab	0/254	0/254	0/51	0/51
**Ilowite et al. 2009[55]**	Anakinra	0/25	0/25	0/25	0/25
**Khanna et al. 2016[57] Khanna et al. 2018 (faSScinate)[58]**	Tocilizumab	13/74	Not reported	3/44	Not reported
**Kivitz et al. 2014[59]**	Tociliuzumab	12/437	0/437	5/218	0/218
**Kivitz et al. 2018[60] (MEASURE 4)**	Secukinumab	8/346	8/346	0/117	1/117
**Lovell et al. 2013[63]**	Rilonacept	0/23	0/23	0/7	0/7
**Maini et al. 2006[64]**	Tocilizumab	7/310	0/310	0/49	0/49
**Martin et al. 2013[65]**	Brodalumab	0/30	0/30	0/10	0/10
**McInnes et al. 2013[67]**	Ustekinumab	5/409	0/409	0/206	0/206
**McInnes et al. 2014[70]**	Secukinumab	1/28	0/28	0/14	0/14

© 2019 Bilal J et al. *JAMA Network Open.*

**McInnes et al. 2017[69]<br>McInnes et al. 2015[68]<br>(FUTURE 2)**	Secukinumab	12/387	17/387	4/98	Not reported
**Mease et al. 2014[72]**	Brodalumab	2/112	0/112	1/55	0/55
**Mease et al. 2015[75]**	Secukinumab	19/404	10/404	1/202	0/202
**Mease et al. 2016 (1)[73]**	Clazakizumab	0/124	0/124	0/41	0/41
**Mease et al. 2016 (2)[76]**	Ixekizumab	3/209	2/209	0/106	0/106
**Mease et al. 2018[71] (FUTURE 5)**	Secukinumab	Not reported	7/822	Not reported	2/332
**Mease et al. 2018[74]<br>Mease et al. 2015[75]<br>Kavanaugh et al. 2017[56]<br>(FUTURE 1)**	Secukinumab	27/726	17/726	1/202	0/202
**Mitha et al. 2013[77]**	Rilonacept	2/166	0/166	0/82	0/82
**Nash et al. 2017[78]**	Ixekizumab	3/245	8/245	0/118	0/118
**Nash et al. 2018[79]**	Secukinumab	Not reported	14/406	Not reported	2/137
**Nishimoto et al. 2009[80] (SATORI)**	Tocilizumab	2/61	Not reported	1/64	Not reported
**Norheim et al. 2012[81]**	Anakinra	1/13	Not reported	0/13	Not reported
**Pavelka et al. 2015[82]**	Brodalumab	1/189	Not reported	0/63	Not reported
**Pavelka et al. 2017[83] (MEASURE 3)**	Secukinumab	7/223	4/223	6/75	0/75
**Quartier et al. 2010[84]**	Anakinra	4/12	2/12	0/12	0/12
**Ritchlin et al. 2014[85]**	Ustekinumab	2/238	1/238	1/104	0/104
**Rovin et al. 2016[86]**	Sirukumab	8/21	Not reported	0/4	Not reported
**Schumacher et al. 2012 (phase III)[87]**	Rilonacept	1/161	0/161	1/79	0/79
**Scott et al. 2016[107]**	Anakinra	1/79	0/79	2/75	0/75
**Sieper et al. 2015[89]**	Sarilumab	1/250	Not reported	0/50	Not reported
**Smolen et al. 2008[93]**	Tocilizumab	9/418	0/418	2/204	0/204
**Smolen et al. 2014[94]**	Sirukumab	7/147	Not reported	1/30	Not reported
**Smolen et al. 2017[92]**	Guselkumab + Ustekinumab	3/218	0/218	1/55	0/55
**Stone et al. 2017[95]**	Tocilizumab	9/150	Not reported	7/101	Not reported
**Sundy et al. 2014[96]**	Rilonacept	9/985	0/985	2/330	0/330
**Tahir et al. 2017[97]**	Secukinumab	30/601	Not reported	3/214	Not reported
**Takeuchi et al. 2017[98]**	Sirukumab	49/1325	Not reported	10/556	Not reported
**Terkeltub et al. 2013[99]**	Rilonacept	0/149	0/149	0/76	0/76
**Tlustochowicz et al. 2016[100]**	Secukinumab	5/177	0/177	1/44	0/44
**Van der Heijde et al. 2018[101]**	Ixekizumab	2/164	0/164	0/87	0/87
**van Vollenhoven et al. 2018[102]**	Ustekinumab	2/60	0/60	0/42	0/42
**Villiger et al. 2016[103]**	Tocilizumab	1/20	0/20	0/10	0/10
**Weinblatt et al. 2015[104]**	Clazakizumab	12/298	0/298	2/61	0/61
**Yazici et al. 2012[105]**	Tocilizumab	12/409	0/409	1/205	0/205

eTable 2: Studies included in assessment of risk of opportunistic infections

Study	Drug	Opportunistic Infections in Treatment Group<br>**Events/Subjects**	**Opportunistic Infections in Control Group<br>Events/Subjects**	**Type**
**Baeten et al. 2015[33] Marzo-Ortega et al. 2017[66] (MEASURE 2)**	Secukinumab	2/211	0/74	Oral candida (2)
**Baeten et al. 2015[33] Braun et al. 2017[38] Baraliakos et al. 2018[35] (MEASURE 1)**	Secukinumab	2/360	0/122	Oral candida
**Burmester et al. 2017[40]**	Tocilizumab	3/870	0/287	Primary tuberculosis (2)
**Deodhar et al. 2018[45]**	Guselkumab	1/129	0/49	Esophageal candida
**Fleischmann et al. 2017[47] (TARGET)**	Sarilumab	2/365	1/181	Non-disseminated *Herpes zoster* infection
**Genovese et al. 2008[53]**	Tocilizumab	1/1220	0/415	*Mycobacterium avium* intracellular infection
**Genovese et al. 2015[51] (MOBILITY)**	Sarilumab	7/855	2/427	8 cases of localized *Herpes zoster* (6 in the sarilumab group, 2 in the placebo group), and 1 case of fungal (Candida)
**Ilowite et al. 2009[55]**	Anakinra	3/25	0/25	Atypical mycobacterial infection, histoplasmosis, and candida esophagitis
**Kivitz et al. 2014[59]**	Tociliuzuma	0/437	1/219	Not reported
**Kremer et al. 2011[61] Kremer et al. 2016[62]**	Tocilizumab	1/1149	0/392	Not reported
**McInnes et al. 2017[69] McInnes et al. 2015[68] (FUTURE**	Secukinumab	11/387	0/98	Oral candida (10)<br>Esophageal candida (1)
**Mease et al. 2018[74] (FUTURE 5)**	Secukinumab	4/822	1/332	Oral candida (5)
**Mease et al. 2017 (2)[76]**	Ixekizumab	2/209	0/106	Esophageal candida and oral
**Nash et al. 2017[78]**	Ixekizumab	4/245	0/118	Oral candida (4)

© 2019 Bilal J et al. *JAMA Network Open.*

eTable 3: Studies included in assessment of risk of malignancy

		Malignancy in Treatment Group		Malignancy in Control Group	
**Study**	**Drug**	**Events/ Subjects**	**Type of Malignancy**	**Events/ Subjects**	**Type of Malignancy**
**Aletaha et al. 2017 (SIRROUND- T)[2]**	Sirukumab	9/834	2 SCC, 2 BCC, 1 rectal adenocarcinoma, 1 lymphoproliferative disorder, 1 oropharyngeal SCC, 1 invasive ductal	1/294	Not specified
**Alten et al. 2011[30]**	Canakinumab	0/204	Not applicable	0/70	Not applicable
**Baek et al. 2017[31]**	Tocilizumab	1/89	1 lung adenocarcinoma	0/51	Not applicable
**Baeten et al. 2015[32] Marzo-Ortega et al. 2017[66] (MEASURE 2)**	Secukinumab	2/211	1 malignant melanoma, 1 unspecified	0/74	Not applicable
**Ben-Zvi et al. 2017[36]**	Anakinra	0/12	Not applicable	0/13	Not applicable
**Baeten et al. 2015[33] Braun et al. 2017[38] Baraliakos et al. 2018[35]**	Secukinumab	5/360	1 gastrointestinal stromal	1/122	Unspecified
**Brunner et al. 2015[39]**	Tocilizumab	0/82	Not applicable	0/81	Not applicable
**Burmester et al. 2017[40]**	Tocilizumab	7/1107	2 endometrial, 1 hepatocellular, 1 metastatic bronchial, 1 renal cell, 1 breast, 1 lung	3/282	1 breast, 1 colon,1 renal cell cancer
**Cohen et al. 2002[42]**	Anakinra	1/345	1 breast cancer	1/74	1 large cell lung cancer
**Deodhar et al. 2018[45]**	Guselkumab	1/129	1 BCC	0/49	Not applicable
**Deodhar et al. 2018 (COAST-**	Ixekizumab	1/212	1 acute promyelocytic leukemia	0/104	Not applicable
**Fleischmann et al. 2003[48]**	Anakinra	4/1116	1 metastatic melanoma, 1 colon, 1 uterine, 1 BCC skin	5/283	1 BCC skin, 1 SCC skin, 1 hodgkins lymphoma, 1 bladder, 1 SCC lung
**Fleischmann et al. 2017**	Sarilumab	2/365	1 RCC, 1 skin carcinoma	1/181	ureter carcinoma
**Genovese et al. 2014 (1)[50]**	Olokizumab +	1/176	1 BCC skin	0/55	Not applicable
**Genovese et al. 2014 (2)[49]**	Secukinumab	3/187	1 ovarian, 1 lung, 1 BCC skin	0/50	Not applicable
**Genovese et al. 2014 (3)[52]**	Ixekizumab	4/330	2 breast, 1 TCC bladder, 1 soft tissue	0/118	Not applicable
**Genovese et al. 2015**	Sarilumab	7/855	Not specified	1/427	Not specified
**Huizinga et al. 2014[54]**	Sarilumab	1/254	1 plasmacytoma	2/51	BCC, SCC
**Ilowite et al. 2009[55]**	Anakinra	0/25	Not applicable	0/25	Not applicable
**Khanna et al. 2016[57] Khanna et al. 2018[58] (faSScinate)**	Tocilizumab	2/74	Not specified	0/44	Not specified
**Kivitz et al. 2014[59]**	Tocilizumab	3/437	1 BCC Skin, 1 pancreatic, 1 renal Cell	0/219	Not applicable
**Kremer et al. 2011[61] Kremer et al. 2016[62]**	Tocilizumab	40/1149	10 BCC, 9 SCC, 2 unknown prostate, breast, lung adenocarcinoma	1/392	1 breast
**Lovell et al. 2013[63]**	Rilanocept	0/23	Not applicable	0/7	Not applicable

© 2019 Bilal J et al. *JAMA Network Open.*

**McInnes et al. 2017[69] McInnes et al. 2015[68] (FUTURE 2)**	Secukinumab	10/387	4 BCC, 3 SCC skin, 1 SCC tongue, 1 bowen disease, 1 throat Ca	0/98	Not applicable
**Mease et al. 2014[72]**	Bordalumab	3/112	1 breast, 1 metastatic lung,1 melanoma	0/55	Not applicable
**Mease et al. 2016 (1)[73]**	Clazakizumab	0/ 124	Not applicable	2/41	1 TCC bladder, 1 prostate
**Mease et al. 2016 (2)[76]**	Ixekizumab	1/209	1 malignancy (not specified)	1/106	1 malignancy
**Mease et al. 2018 (FUTURE 5)[71]**	Secukinumab	2/822	1 melanoma<br>1 bladder Ca	0/332	Not applicable
**Mease et al. 2018[74] Mease et al. 2015[75] Kavanaugh et al. 2017 (FUTURE 1)[56]**	Secukinumab	6/587	3 BCC, 2 prostate, 1 pleomorphic adenoma of salivary gland	1/202	1 Breast
**Nash et al. 2017[78]**	Ixekizumab	2/245	1 Prostate, 1 BCC Skin	0/118	Not applicable
**Nash et al. 2018[79]**	Secukinumab	6/406	Not specified	0/137	Not applicable
**Pavelka et al. 2017 (MEASURE 3)[83]**	Secukinumab	2/223	Not specified	0/75	Not applicable
**Ritchlin et al. 2014[85]**	Ustekinumab	2/238	1 SCC skin, 1 breast	0/104	Not applicable
**Rovin et al. 2016[86]**	Sirukumab	0/21	Not applicable	0/4	Not applicable
**Schumacher et al. (phase II) 2012[88]**	Rilanocept	1/41	1 prostate	0/42	Not applicable
**Schumacher et al. (phase III) 2012[87]**	Rilanocept	1/161	1 SCC skin	0/79	Not applicable
**Sieper et al. 2014[91]**	Tocilizumab	0/51	Not applicable	0/51	Not applicable
**Smolen et al. 2008[93]**	Tocilizumab	0/418	Not applicable	1/204	1 skin SCC
**Smolen et al. 2017[92]**	Ustekinumab + Guselkumab	1/218	1 SCC lung	0/55	Not applicable
**Stone et al. 2017[95]**	Tocilizumab	0/149	Not applicable	2/101	Not specified
**Takeuchi et al. 2017[98]**	Sirukumab	7/1325	Not specified	2/556	Not specified
**Van der Heijde et al. 2018[101]**	Ixekizumab	0/164	Not applicable	0/87	Not applicable
**van Vollenhoven et al. 2018[102]**	Ustekinumab	0/60	Not applicable	0/42	Not applicable
**Weinblatt et al. 2015[104]**	Clazakizumab	0/298	Not applicable	0/61	Not applicable
**Yazici et al. 2010[105]**	Tocilizumab	3/409	1 conjunctival malignancy, 1 BCC skin, 1 nonmelanoma skin	3/205	1 SCC Skin, 1 DLBC lymphoma, 1 renal cell

Abbreviations: SCC, squamous cell carcinoma; BCC, basal cell carcinoma

© 2019 Bilal J et al. *JAMA Network Open.*

eTable 4: Grading of Recommendations Assessments, Development and Evaluation (GRADE) assessment of the strength of evidence

Question: To assess the risk of serious infections, opportunistic infections, and malignancy with use interleukin inhibitors vs placebo
Setting: Patients with rheumatic diseases

Certainty assessment							№ of patients		Effect		Certainty	Importance
№ of studies	Study design	Risk of bias	Inconsistency	Indirectness	Imprecision	Other considerations	IL inhibitors	Placebo	Relative (95% CI)	Absolute (95% CI)		
**Serious Infections (follow up: median 24 weeks)**												
69	randomised trials	not serious [a]	not serious	not serious	not serious	none	486/17177 (2.8%)	96/7059 (1.4%)	**OR 1.97** (1.58 to 2.44)	**13 more per 1,000** (from 8 more to 19 more)	⨁⨁⨁⨁ HIGH	CRITICAL
**Malignancy (follow up: median 28 weeks)**												
45	randomised trials	not serious	not serious	not serious	serious [b]	none	141/15244 (0.9%)	28/5821 (0.5%)	**OR 1.51** (1.05 to 2.18)	**2 more per 1,000** (from 0 fewer to 6 more)	⨁⨁⨁◯ MODERATE	IMPORTANT
**Opportunistic Infections (follow up: median 54 weeks)**												
15	randomised trials	not serious	not serious	not serious	serious [b]	none	43/7153 (0.6%)	5/2845 (0.2%)	**OR 2.35** (1.09 to 5.05)	**2 more per 1,000** (from 0 fewer to 7 more)	⨁⨁⨁◯ MODERATE	IMPORTANT

CI: Confidence interval; **OR:** Odds ratio

Explanations

a. While the Publication bias was suspected, the adjusted effect size using trim and fill method was similar.
b. Imprecision caused by the small number of events; which caused wide CIs with lower boundaries close to the null effect.

The certainty in evidence is rated as high for the outcome of serious infections for the following reasons: (i) the evidence was derived from RCTs, (ii) the meta-analytic effect estimates were precise, (iii) the results are consistent (heterogeneity was low or moderate across studies); and (iv) the vast majority of the RCTs included in our study are characterized by low or unclear RoB, as assessed with the Cochrane Collaboration's tool. While the Publication bias was suspected, the adjusted effect size using trim and fill method was similar. On the other hand, the certainty of evidence was rated down to "moderate" for the outcomes of opportunistic infections and malignancy due to imprecision caused by the small number of events; which caused wide CIs with lower boundaries close to the null effect.

© 2019 Bilal J et al. *JAMA Network Open.*

eAppendix. Specific Search Strategy

Ovid

Database(s): EBM Reviews - Cochrane Central Register of Controlled Trials October 2018, EBM Reviews - Cochrane Database of Systematic Reviews 2005 to November 21, 2018, Embase 1974 to 2018 November 29, Ovid MEDLINE(R) and Epub Ahead of Print, In-Process & Other Non-Indexed Citations and Daily 1946 to November 29, 2018

Search Strategy:

#	Searches	Results
1	anakinra/	6208
2	canakinumab/	2340
3	rilonacept/	779
4	tocilizumab/	8859
5	clazakizumab/	123
6	secukinumab/	2125
7	ixekizumab/	1027
8	brodalumab/	704
9	ustekinumab/	5790
10	guselkumab/	279
11	tildrakizumab/	185
12	olokizumab/	73
13	(Anakinra or "anti-IL-1 beta monoclonal antibod*" or "anti-IL-1beta monoclonal antibod*" or "anti-interleukin 1 beta monoclonal antibod*" or "anti-interleukin 1beta monoclonal antibod*" or Brodalumab or Canakinumab or Clazakizumab or Guselkumab or "IL-1 receptor antagonist*" or "IL-1 receptor block*" or "IL-12" or "IL-17" or "IL-17A" or "IL-1b inhibitor*" or "IL-23p19" or "IL-23p40" or "IL-6" or "IL-6 receptor antibod*" or "interleukin 1 receptor antagonist*" or "interleukin 1 receptor block*" or "interleukin-12" or "interleukin-17" or "interleukin-17A" or "interleukin-1b inhibitor*" or "interleukin-23p19" or "interleukin-23p40" or "interleukin-6" or "interleukin-6 receptor antibod*" or Ixekizumab or olokizumab or Rilonacept or Secukinumab or Tildrakizumab or Tocilizumab or Ustekinumab).ti,ab,hw,kw.	495424
14	or/1-13	495424
15	exp Rheumatic Diseases/	446930
16	(("Eosinophilic granulomatos*" and polyangiitis) or ((small or medium) adj3 arter*) or (Aortitis and Cogans syndrome) or (Aortitis and spondylarthropath*) or (Granulomatos* and polyangiitis) or (Vasculitis and ("rheumatoid arthritis" or "systemic lupus erythematosus" or "Sjögrens syndrome")) or "ANCA-associated vasculitis" or "Ankylosing spondylitis" or "anti-GBM antibody disease" or "Antiglomerular basement membrane antibody disease" or "Antiphospholipid Syndrome" or "Behets disease" or "Churg-Strauss" or "Cryoglobulinemic vasculitis" or "Cryopyrin-Associated Autoinflammatory Syndrome" or "Cryopyrin-associated periodic syndrome*" or Dermatomyositis or "Drug-induced vasculitis" or "Enteropathic arthritis" or "Familial cold autoinflammatory syndrome*" or "Familial cold auto-inflammatory syndrome*" or "Familial Mediterranean Fever" or Fibromyalgia or "Giant cell arteritis" or "Goodpastures syndrome" or Gout or "Henoch-Schnlein" or "Hyperimmunoglobulin D Syndrome" or Hypermobility or "IgA Vasculitis" or "Isolated aortitis" or JIA or "Juvenile arthritis" or "Juvenile idiopathic arthritis" or "Juvenile scleroderma" or "Kawasaki disease" or "Localized Scleroderma" or Lupus or "Lyme Disease" or "Metabolic Myopath*" or "Microscopic polyangiitis" or "Muckle Wells Syndrome" or "Multicentric Castlemans disease" or Osteoarthritis or "Pagets Disease of Bone" or "Periodic Fever Aphthous Stomatitis Pharyngitis Adenitis" or PFAPA or "Plaque psoriasis" or "Polyarteritis nodosa" or "Polymyalgia Rheumatica" or "Primary angiitis of the central nervous system" or "psoriatic arthritis" or "Raynauds Phenomenon" or "Reactive arthritis" or "Relapsing polychondritis" or "rheumatic disease*" or "Rheumatoid Arthritis" or "rheumatologic disease*" or "Schnitzler syndrome" or Scleroderma or "seronegative spondylarthropath*" or "Sjgrens Syndrome" or Spondyloarthritis or spondyloarthros* or "Stills disease" or "Systemic Lupus Erythematosus" or "Systemic sclerosis" or "Takayasus arteritis" or TRAPS or "Tumor Necrosis Factor Receptor Associated Periodic Syndrome" or "Undifferentiated spondyloarthropath*" or "Wegeners granulomatosis").ti,ab,hw,kw.	1114533
17	15 or 16	1165102
18	14 and 17	55477
19	exp meta analysis/	248588
20	exp Meta-Analysis as Topic/	56373
21	exp "systematic review"/	187127
22	exp controlled study/	6485469
23	exp Randomized Controlled Trial/	998458
24	exp triple blind procedure/	199
25	exp Double-Blind Method/	432679
26	exp Single-Blind Method/	77694
27	exp latin square design/	361
28	exp Placebos/	384747
29	exp Placebo Effect/	10926
30	exp clinical trial/	2161049
31	clinical study/	154313
32	((meta adj analys*) or metaanalys* or (systematic* adj3 review*) or (control* adj3 study) or (control* adj3 trial) or (randomized adj3 study) or (randomized adj3 trial) or (randomised adj3 study) or (randomised adj3 trial) or "pragmatic clinical trial" or (random* adj1 allocat*) or (doubl* adj blind*) or (doubl* adj mask*) or (singl* adj blind*) or (singl* adj mask*) or (tripl* adj blind*) or (tripl* adj mask*) or (trebl* adj blind*) or (trebl* adj mask*) or "latin square" or placebo* or nocebo* or (intervention* adj2 study) or (intervention* adj2 trial) or "clinical study" or "clinical trial" or trial or (("phase 0" or "phase 1" or "phase I" or "phase 2" or "phase II" or "phase 3" or "phase III" or "phase 4" or "phase IV") adj5 (trial or study))).mp,pt.	12207161
33	or/19-32	12223858
34	18 and 33	25460

© 2019 Bilal J et al. *JAMA Network Open.*

35	(random* and placebo).ti,ab,hw,kw.	551795
36	34 and 35	4109
37	limit 36 to (editorial or erratum or note or addresses or autobiography or bibliography or biography or blogs or comment or dictionary or directory or interactive tutorial or interview or lectures or legal cases or legislation or news or newspaper article or overall or patient education handout or periodical index or portraits or published erratum or video-audio media or webcasts) [Limit not valid in CCTR,CDSR,Embase,Ovid MEDLINE(R),Ovid MEDLINE(R) Daily Update,Ovid MEDLINE(R) In-Process,Ovid MEDLINE(R) Publisher; records were retained]	53
38	from 37 keep 2-11	10
39	(36 not 37) or 38	4066
40	remove duplicates from 39	2665

© 2019 Bilal J et al. *JAMA Network Open.*

Scopus

1 TITLE-ABS-KEY(Anakinra OR "anti-IL-1 beta monoclonal antibod*" OR "anti-IL-1beta monoclonal antibod*" OR "anti-interleukin 1 beta monoclonal antibod*" OR "anti-interleukin 1beta monoclonal antibod*" OR Brodalumab OR Canakinumab OR Clazakizumab OR Guselkumab OR "IL-1 receptor antagonist*" OR "IL-1 receptor block*" OR "IL-12" OR "IL-17" OR "IL-17A" OR "IL-1b inhibitor*" OR "IL-23p19" OR "IL-23p40" OR "IL-6" OR "IL-6 receptor antibod*" OR "interleukin 1 receptor antagonist*" OR "interleukin 1 receptor block*" OR "interleukin-12" OR "interleukin-17" OR "interleukin-17A" OR "interleukin-1b inhibitor*" OR "interleukin-23p19" OR "interleukin-23p40" OR "interleukin-6" OR "interleukin-6 receptor antibod*" OR Ixekizumab OR olokizumab OR Rilonacept OR Secukinumab OR Tildrakizumab OR Tocilizumab OR Ustekinumab)

2 TITLE-ABS-KEY(("Eosinophilic granulomatos*" and polyangiitis) OR ((small or medium) W/3 arter*) OR (Aortitis and Cogans syndrome) OR (Aortitis and spondylarthropath*) OR (Granulomatos* and polyangiitis) OR (Vasculitis and ("rheumatoid arthritis" or "systemic lupus erythematosus" or "Sjögrens syndrome")) OR "ANCA-associated vasculitis" OR "Ankylosing spondylitis" OR "anti-GBM antibody disease" OR "Antiglomerular basement membrane antibody disease" OR "Antiphospholipid Syndrome" OR "Behets disease" OR "Churg-Strauss" OR "Cryoglobulinemic vasculitis" OR "Cryopyrin-Associated Autoinflammatory Syndrome" OR "Cryopyrin-associated periodic syndrome*" OR Dermatomyositis OR "Drug-induced vasculitis" OR "Enteropathic arthritis" OR "Familial cold autoinflammatory syndrome*" OR "Familial cold auto-inflammatory syndrome*" OR "Familial Mediterranean Fever" OR Fibromyalgia OR "Giant cell arteritis" OR "Goodpastures syndrome" OR Gout OR "Henoch-Schnlein" OR "Hyperimmunoglobulin D Syndrome" OR Hypermobility OR "IgA Vasculitis" OR "Isolated aortitis" OR JIA OR "Juvenile arthritis" OR "Juvenile idiopathic arthritis" OR "Juvenile scleroderma" OR "Kawasaki disease" OR "Localized Scleroderma" OR Lupus OR "Lyme Disease" OR "Metabolic Myopath*" OR "Microscopic polyangiitis" OR "Muckle Wells Syndrome" OR "Multicentric Castlemans disease" OR Osteoarthritis OR "Pagets Disease of Bone" OR "Periodic Fever Aphthous Stomatitis Pharyngitis Adenitis" OR PFAPA OR "Plaque psoriasis" OR "Polyarteritis nodosa" OR "Polymyalgia Rheumatica" OR "Primary angiitis of the central nervous system" OR "psoriatic arthritis" OR "Raynauds Phenomenon" OR "Reactive arthritis" OR "Relapsing polychondritis" OR "rheumatic disease*" OR "Rheumatoid Arthritis" OR "rheumatologic disease*" OR "Schnitzler syndrome" OR Scleroderma OR "seronegative spondylarthropath*" OR "Sjgrens Syndrome" OR Spondyloarthritis OR spondyloarthros* OR "Stills disease" OR "Systemic Lupus Erythematosus" OR "Systemic sclerosis" OR "Takayasus arteritis" OR TRAPS OR "Tumor Necrosis Factor Receptor Associated Periodic Syndrome" OR "Undifferentiated spondyloarthropath*" OR "Wegeners granulomatosis")

3 TITLE-ABS-KEY((meta W/1 analys*) OR metaanalys* OR (systematic* W/3 review*) OR (control* W/3 study) OR (control* W/3 trial) OR (randomized W/3 study) OR (randomized W/3 trial) OR (randomised W/3 study) OR (randomised W/3 trial) OR "pragmatic clinical trial" OR (random* W/1 allocat*) OR (doubl* W/1 blind*) OR (doubl* W/1 mask*) OR (singl* W/1 blind*) OR (singl* W/1 mask*) OR (tripl* W/1 blind*) OR (tripl* W/1 mask*) OR (trebl* W/1 blind*) OR (trebl* W/1 mask*) OR "latin square" OR placebo* OR nocebo* OR (intervention* W/2 study) OR (intervention* W/2 trial) OR "clinical study" OR "clinical trial" OR trial OR (("phase 0" or "phase 1" or "phase I" or "phase 2" or "phase II" or "phase 3" or "phase III" or "phase 4" or "phase IV") W/5 (trial or study)))

4 TITLE-ABS-KEY(random* and placebo)

5 1 and 2 and 3 and 4

6 DOCTYPE(ed) OR DOCTYPE(bk) OR DOCTYPE(er) OR DOCTYPE(no) OR DOCTYPE(sh)

7 5 and not 6

8 INDEX(embase) OR INDEX(medline) OR PMID(0* OR 1* OR 2* OR 3* OR 4* OR 5* OR 6* OR 7* OR 8* OR 9*)

9 7 and not 8

© 2019 Bilal J et al. *JAMA Network Open.*
